# Integrated omics-analysis reveals Wnt-mediated NAD^+^ metabolic reprogramming in cancer stem-like cells

**DOI:** 10.18632/oncotarget.10432

**Published:** 2016-07-06

**Authors:** Jueun Lee, Hyun Jung Kee, Soonki Min, Ki Cheong Park, Sunho Park, Tae Hyun Hwang, Do Hyun Ryu, Geum-Sook Hwang, Jae-Ho Cheong

**Affiliations:** ^1^ Integrated Metabolomics Research Group, Western Seoul Center, Korea Basic Science Institute, Seoul 03760, Republic of Korea; ^2^ Department of Chemistry, Sungkyunkwan University, Suwon 16419, Republic of Korea; ^3^ Department of Surgery, Yonsei University College of Medicine, Seoul 03722, Republic of Korea; ^4^ Department of Clinical Sciences, University of Texas Southwestern Medical Center, Dallas, TX 75390, USA; ^5^ Department of Life Science, Ewha Womans University, Seoul 03760, Republic of Korea; ^6^ Department of Biochemistry & Molecular Biology, Yonsei University College of Medicine, Seoul 03722, Republic of Korea; ^7^ BK21 PLUS Projects for Medical Science, Yonsei University College of Medicine, Seoul 03722, Republic of Korea; ^8^ Open NBI Convergence Technology Research Laboratory, Department of Surgery, Yonsei University College of Medicine, Seoul 03722, Republic of Korea

**Keywords:** calcium signaling, cancer stem cells, integrated analysis, metabolic reprogramming, Wnt signaling

## Abstract

Abnormal tumor cell metabolism is a consequence of alterations in signaling pathways that provide critical selective advantage to cancer cells. However, a systematic characterization of the metabolic and signaling pathways altered in cancer stem-like cells (CSCs) is currently lacking. Using nuclear magnetic resonance and mass spectrometry, we profiled the whole-cell metabolites of a pair of parental (P-231) and stem-like cancer cells (S-231), and then integrated with whole transcriptome profiles. We identified elevated NAAD^+^ in S-231 along with a coordinated increased expression of genes in Wnt/calcium signaling pathway, reflecting the correlation between metabolic reprogramming and altered signaling pathways. The expression of CD38 and ALP, upstream NAAD^+^ regulatory enzymes, was oppositely regulated between P- and S-231; high CD38 strongly correlated with NAADP in P-231 while high ALP with NAAD^+^ levels in S-231. Antagonizing Wnt activity by dnTCF4 transfection reversed the levels of NAAD^+^ and ALP expression in S-231. Of note, elevated NAAD^+^ caused a decrease of cytosolic Ca^2+^ levels preventing calcium-induced apoptosis in nutrient-deprived conditions. Reprograming of NAD^+^ metabolic pathway instigated by Wnt signaling prevented cytosolic Ca^2+^ overload thereby inhibiting calcium-induced apoptosis in S-231. These results suggest that “oncometabolites” resulting from cross talk between the deranged core cancer signaling pathway and metabolic network provide a selective advantage to CSCs.

## BACKGROUND

Malignant progression requires coordinated adaptation of cellular metabolism that alters the physiology of transformed cells providing selective advantages [1]. In the tumor metabolic microenvironment, which is continuously reshaped during tumor progression and provides selective pressure, tumor cells can acquire adaptive characteristics, including resistance to apoptosis, invasion, and metastasis [2–4]. These acquired phenotypes share characteristics with tumor initiating cells or cancer stem-like cells (CSCs). The acquisition of cellular phenotypes in the stressful microenvironment is mediated by transcriptional reprogramming that in turn leads to changes in signaling pathways [3, 5]. It is well established that the clinical outcomes of cancers frequently depend on the occurrence of metastasis and resistance to therapy, which might be a contribution of CSCs. Thus, comprehensive understanding of molecular physiology of CSCs is indispensible for developing promising anti-cancer strategy.

With the recent advent of high-throughput “-omics” technologies and the great interest in cancer pathobiology, unprecedented opportunities to understand the mechanisms underlying cancer pathogenesis have arisen. Omics technologies, such as genomics, transcriptomics, proteomics, and metabolomics, are also described as global molecular profiling, and allow for the collection and quantification of pools of biological molecules that provide valuable information on biological functions and dynamics at multi-dimensional levels [6]. The integration of these techniques allows us to understand complex biological systems such as cancer and gain a broader picture of systems behavior.

Metabolomics is a top-down platform in the field of systems biology that focuses on the dynamic changes of metabolites in a given biological context. The metabolic phenotypes of tumor cells are controlled by intrinsic genetic alterations and responses to alterations in the tumor microenvironment. Thus, a metabolic discrepancy in cancer phenotypes sheds light on diverse underlying biological events [7]. Recently, several metabolomics approaches have been applied to characterize the metabolic phenotypes of CSCs using both untargeted and targeted techniques [8–10]. In addition, metabolomics studies integrated with proteomics were successfully applied to *in vitro* models for CSCs, and revealed that cancer metabolism was reprogrammed by the regulation of energetic and glycolytic fluxes [11–13]. However, although metabolic and signaling reprogramming in the tumor microenvironment is thought to play an essential role in the emergence of CSCs, a systematic and integrated characterization of the metabolic pathways active in CSCs is currently lacking. Further, the signaling pathways that are related to these metabolic alterations remain undefined. To systematically characterize CSCs metabolic and transcriptional reprogramming, we used nuclear magnetic resonance (NMR) and liquid chromatography (LC)-tandem mass spectrometry (MS/MS) to create cellular metabolome profiles and conducted genome-wide transcriptome profiling of CSCs.

The integrated metabolome and transcriptome analyses revealed that the NAD^+^ metabolic pathway was altered by the Wnt core signaling pathway, resulting in an increased level of NAAD^+^ in CSCs. Importantly, elevated NAAD^+^ curbs cytosolic Ca^2+^ overload thereby preventing calcium-induced apoptosis in nutrient-deprived conditions. These results suggest that the deranged core cancer signaling pathway cross-talks with metabolic pathways and the resultant “oncometabolites” might thus provide a selective advantage to CSCs.

## RESULTS

### NAAD^+^ increases in chronic metabolic stress induced stem-like cancer cells

We recently reported that chronic metabolic stress induces cancer stem-cell like phenoconversion mediated by Wnt pathway activation [14]. Intriguingly, these CSCs exhibited enhanced survival capability in prolonged glucose starvation conditions, suggesting the acquisition of cellular processes that negate metabolic stress induced cell death [14].

**Figure 1:**
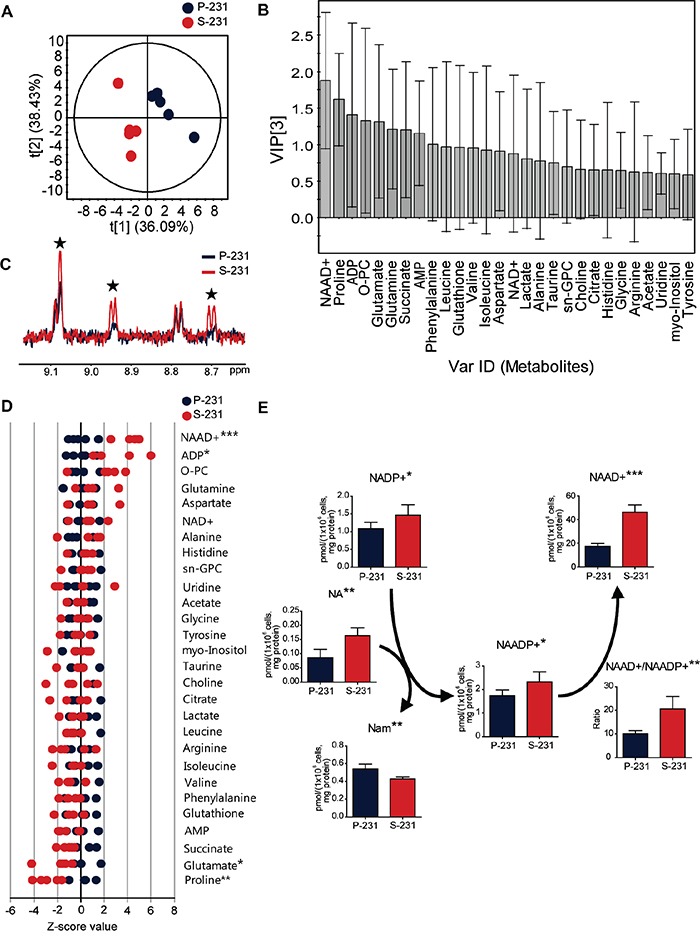
NAAD^+^ is a key metabolite representing stem-like cancer cell phenotypes **A.** PLS-DA score plot discriminating parental (blue) and selected (red) cells. The fit of the model to the data is described by *R^2^X* (0.815), *R^2^Y* (0.988) and *Q^2^* (0.840). The ellipse represents the 95% confidence region for Hotelling's *T*^2^. **B.** VIP plot from PLS-DA showing the important metabolites. Metabolites with a high VIP (VIP > 1) and low P value (*p* < 0.05) was considered as key compounds responsible for the separation between groups in the PLS-DA score plot. **C.** Enhancement of NAAD^+^ signals in S-231 according to ^1^H NMR spectra. Blue and red spectra are representative of parental cancer cells and selected cancer stem-like cells, respectively. Black stars indicate NAAD^+^ identities. **D.** Z-score plot highlighting higher relative metabolite levels in parental (*left*) and selected (*right*) cells. Each point indicates one metabolite in one sample. **E.** Alterations in NAAD^+^-related metabolites using LC-MS/MS analysis. Concentration is expressed in pmol per 1 × 10^6^ cells and corrected by protein (mg). Data expressed as means ± standard deviation (S.D.). P-231, n = 5; S-231, n = 5. *, ** and *** indicate *p* < 0.05, < 0.01 and < 0.001, respectively. ADP, adenosine diphosphate; AMP, adenosine monophosphate; sn-GPC, glycero-3-phosphocholine; O-PC, O-phosphocholine; NAAD^+^, nicotinic acid adenine dinucleotide. NAADP^+^, nicotinic acid adenine dinucleotide phosphate; NADP^+^, nicotinamide adenine dinucleotide phosphate (oxidized from); Nam, nicotinamide; NA, nicotinic acid.

Some cancer cells increase their survival capacity under conditions of glucose deprivation [15, 16] by circumventing apoptotic signals. Indeed, CSCs displayed remarkably higher viability than did non-CSCs, which rapidly underwent apoptotic cell death in glucose deprivation [14] (Figure S1). We hypothesized that CSCs could have undergone a metabolic reprogramming associated with anti-apoptotic capability upon chronic metabolic stress arising from glucose deprivation. To investigate the potential metabolic alterations in CSCs, NMR- and MS-based metabolite profiling was performed in MDA-MB 231 parental cells (P-231) and stem-like cancer cells (S-231). First, to identify the important metabolites discriminating P-231 and S-231, a partial least squares discriminant analysis (PLS-DA) model was generated from target metabolite profiles based on ^1^H NMR spectroscopy. The PLS-DA score plot indicated a clear discrepancy between P-231 and S-231 (Figure 1A). The reliability of the PLS-DA model was validated using a 100-fold repeated permutation test (Figure S2). To investigate which metabolite was most important for the differences in response between P-231 and S-231, we evaluated the variable importance in the projection (VIP) values of each metabolite identified by PLS-DA. Metabolites, including ADP, glutamate, proline, and NAAD^+^, contributed to both groups with high VIP values (> 1.0) and significance (*p* < 0.05; Figure 1B). Representative ^1^H NMR spectra with 28 labeled metabolites from P-231 and S-231 are illustrated in Figure S3. The identities of the examined metabolites, concentrations, fold changes, and P values are listed in Table S1. Overlaying the ^1^H NMR spectra from the various cell extracts also indicated enhancement of the NAAD^+^ peak (Figure 1C). Based on the Z-score plot, the most highly elevated metabolites were ADP and NAAD^+^, whereas glutamate and proline were less abundant in S-231 (Figure 1D). NAAD^+^ was therefore considered one of the key discriminatory factors between the two groups. In fact, S-231 contained approximately two fold higher NAAD^+^ concentrations than P-231 (*p* < 0.001; Table S1).

To further verify changes in NAAD^+^-related metabolism between P-231 and S-231, LC-tandem MS was utilized to quantify 10 metabolites. Representative ion chromatograms from cell extracts are illustrated in Figure S4. Significantly altered metabolites within the NAAD^+^ pathway are presented in Figure 1E. We found that both NADP^+^ and NAADP^+^ levels were higher in S-231 than in P-231 and that the concentration of NA, necessary for the conversion of NADP^+^ to NAADP^+^, which is then metabolized to Nam, was elevated in S-231 and decreased in P-231. We also noted that higher quantities of NAAD^+^ were detected in S-231 relative to P-231. Furthermore, the ratio of NAAD^+^ to NAADP^+^ significantly increased in S-231. The above results suggest that NAAD^+^ accumulation represents a distinct characteristic of S-231 and is likely induced by alterations in intracellular NAAD^+^ metabolism. Equivalent results were obtained in P-231 and S-231 grown in the absence of glucose (Table S2).

### Transcriptional reprograming of stem-like cancer cells

**Figure 2:**
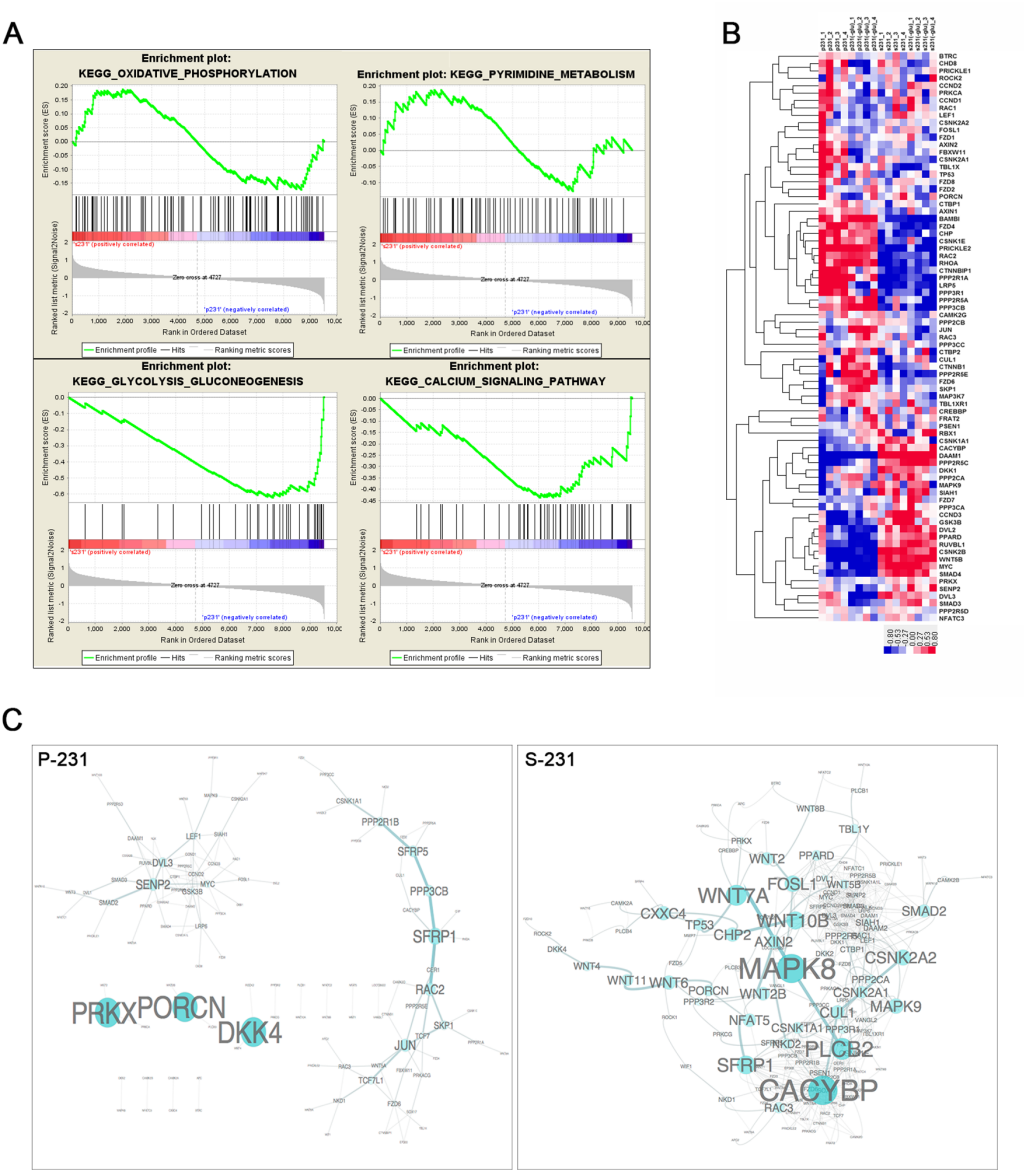
Transcriptional reprograming of stem-like cancer cells **A.** Unsupervised enrichment plots of parental cancer and selected cancer stem-like cells using GSEA (P; parental, S; stem-like cancer cell). Gene set analysis of KEGG pathway identified the functional categories of gene sets that are differentially expressed between parental cancer and selected cancer stem-like cells. The enrichment gene categories of oxidative phosphorylation and pyrimidine metabolism positively correlate with S-231, whereas the enrichment gene categories of glycolysis, gluconeogenesis and the calcium signaling pathway are negatively correlated **B.** Heat map of the Wnt pathway. Hierarchical clustering analysis of genes showed significant differences between P-231 and S-231 in the presence (+) or absence (−) of glucose. In the heat map, red denotes higher relative expression, whereas blue indicates lower relative expression, with the degree of color saturation reflecting the magnitude of the log expression signal. The bottom row represents the median log expression value. **C.** The Wnt sub-network of P-231 versus S-231 in the presence of glucose. We generated network views of the Wnt co-expression networks containing genes that were strongly co-expressed in S-231. Notably, many Wnt pathway member genes showed high co-expression in S-231 but not in P-231. MAPK8 serves as a hub in the network, and many genes are densely networked with each other in the S-231.

Since gene expression programming is largely influenced by signals from the environment, we speculated that stem-like cancer cells would achieve a stem-ness state by transcriptional reprogramming. To elucidate the potential mechanisms that conferred a CSCs phenotype, we performed genome-wide transcriptional profiling of paired P-231 and S-231. A large number of genes were significantly differentially expressed between P-231 and S-231, suggesting that multiple biological processes are reprogrammed in S-231. To identify the biological pathways and processes altered in S-231 as a consequence of differences in gene expression, we performed a gene set enrichment analysis (GSEA). GSEA is especially suited for investigating gene expression data relating to distinct biological processes across two conditions at a gene set level. This analysis identified significantly enriched gene sets that were characteristic of S-231. In the up-regulated gene sets of the S-231 group, 29 were significantly enriched at a nominal p-value < 0.05. Of these, the calcium signaling, oxidative phosphorylation, pyrimidine metabolism, and glycolysis/gluconeogenesis pathways were highly significantly enriched (p value=0.02, FDR q value=0.25) (Figure 2A). From the results of the enrichment analysis of transcriptome profiling, we focused on Wnt signaling which is associated with stem-ness state. Wnt signaling activity is required to promote the survival and self-renewal capability of CSCs [17–20]. We collected Wnt pathway genes from the KEGG database; many of these, notably, overlap member genes of the calcium signaling pathway as well. We then generated a heat map of Wnt signaling (Figure 2B) and computed the Pearson's correlations between pairs of member genes of Wnt pathways to evaluate Wnt co-expression modules for both parental and stem-like cancer cells. We also generated network views of selected Wnt co-expression networks that were strongly co-expressed in S-231 compared to P-231 (Figure 2C). Thus, the results confirmed that S-231 exhibited elevated canonical and non-canonical Wnt pathways activities through transcriptional reprogramming.

### Integrated transcriptomic and metabolomic analyses reveal characteristics of cancer stem-ness by regulating genetic factors and NAAD^+^ metabolism

**Figure 3:**
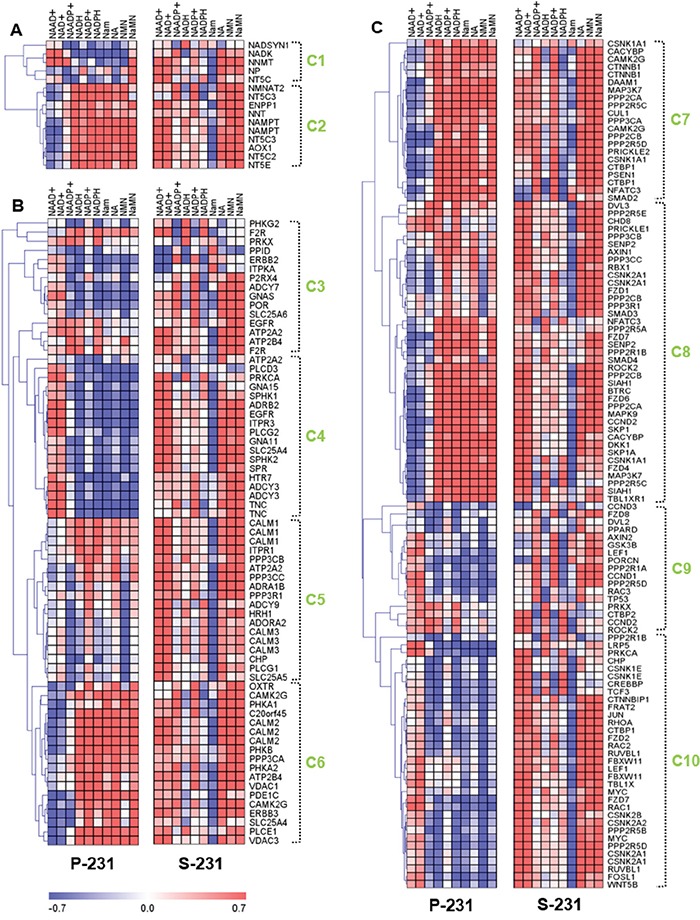
Correlation analysis between transcriptomes and metabolomes reveal characteristics of cancer stemness by regulating genetic factors and NAAD^+^ metabolism **A.** The correlation matrix of nicotinate and nicotinamide metabolism shows the differently associated genes between P-231 and S-231, including *NT5C, ENPP1, NMNAT2, NNT,* and *NT5E*. **B.** Genes from clusters 4, 5 and 6 in the Ca^2+^ signaling pathway show markedly altered correlations between P-231 and S-231. *ATP2A2* and *SLC25A5* exhibit a stronger positive correlation with NAAD^+^ in S-231, whereas ITPKA exhibits a remarkably negative relationship with NAAD^+^ in S-231 compared to P-231. **C.** The correlation matrixes between the Wnt signaling pathway and NAAD^+^ metabolites indicate that *CACYBP, CSNK1A1, CSNK1E, CSNK2B, DAAM1, MYC, PPP2CB, PPP2R5C, SMAD4, SIAH1*, and *WNT5B* are highly associated with NAAD^+^ in S-231. Rows represent NAAD^+^ metabolites detected by LC-MS/MS analysis, and columns represent genes measured by microarray, which are hierarchically clustered. Ten sub-clusters were arbitrarily assigned and are indicated by light green letters (C1 to C10). The Pearson's correlation coefficients range in value from −0.7 to +0.7 and are indicated in red and blue (positive and negative correlation, respectively). NAAD^+^, nicotinic acid adenine dinucleotide; NAADP^+^, nicotinic acid adenine dinucleotide phosphate; NAD^+^, nicotinamide adenine dinucleotide (oxidized form); NADH, nicotinamide adenine dinucleotide (reduced form); NADP^+^, nicotinamide adenine dinucleotide phosphate (oxidized form); NADPH, nicotinamide adenine dinucleotide phosphate (reduced form); Nam, nicotinamide; NMN, nicotinamide mononucleotide; NA, nicotinic acid; NaMN, nicotinic acid mononucleotide.

Gene-metabolite relationships provide valuable clues for finding direct or indirect causes of cancer stem-ness at the molecular level [21, 22]. To directly evaluate the relationship between transcriptome and metabolome expression, we used an integrative Pearson's correlation analysis for selected genes and metabolites. To determine which differentially expressed gene features across P-231 and S-231 would be utilized for further analysis, gene ontology (GO) analysis of transcriptome profiles was used; this identified the Ca^2+^ signaling pathway, nicotinate and nicotinamide metabolism, and the Wnt signaling pathway. Likewise, for differentially represented metabolites, NAAD^+^ and NAAD^+^ intermediates were identified. As a result, a total of 194 genes and 10 metabolites were chosen for downstream analysis to assess the differences between the P-231 and S-231. Ten clusters that were arbitrarily assigned showed distinct correlation trends between the P-231 and S-231 in a hierarchical clustering tree (Figure 3). To further investigate systematic molecular changes, differentially expressed genes (*p* < 0.05) from glucose-deprived cancer cells were mapped in various pathways. Genes determined to be significantly expressed are listed in Table S3.

Analysis of two gene clusters in the nicotinate and nicotinamide metabolic pathway suggested the existence of differences between two independent correlations from P-231 and S-231 (Figure 3A). Genes in cluster 2 were positively associated with NAAD^+^ in S-231, whereas the opposite was true in P-231. The expression levels of these genes were also significantly altered between the two groups. For example, *NT5C* expression was higher in S-231 than in P-231. However, *ENPP1, NMNAT2, NNT*, and *NT5E* were expressed at lower levels in S-231 relative to P-231, indicating a reduction in NAAD^+^ biosynthetic production (Figure S5).

With respect to the Ca^2+^ signaling pathway, clusters 4, 5 and 6 showed markedly altered correlation patterns between P-231 and S-231 (Figure 3B). Notably, NAAD^+^ was more positively associated with clusters 5 and 6 in S-231 than in P-231. Furthermore, *ATP2A2* and *SLC25A5* demonstrated a stronger positive correlation with NAAD^+^ in S-231 than in P-231 and confirmed the positive correlation between NAAD^+^ and the Ca^2+^ signaling pathway in stem-like cancer cells. On the other hand, *ITPKA* exhibited a remarkably negative relationship with NAAD^+^ in S-231, but not in P-231. *ATP2A2, PPID, PRKX*, and *SLC25A5* were significantly higher in the Ca^2+^ signaling pathway from S-231 compared with that from P-231. In contrast, lower levels of *ADCY3, ADCY9, ATP2B4, CLALM3, CHP, GNA11, GNA15, GNAS, ITPKA, ITPR1, PLCD3, PLCG2, PPP3R1*, and *PRKCA* were observed in S-231. The gene-annotated Ca^2+^ signaling pathway indicated that the expression of genes involved in Ca^2+^ secretion into the cytoplasm decreased, whereas genes associated with Ca^2+^ accumulation into the endoplasmic reticulum and mitochondrial excretion were upregulated in S-231 (Figure S6).

With respect to the Wnt signaling pathway, most genes exhibited differential associations with NAAD^+^ metabolites in S-231 compared to those in P-231 (Figure 3C). Clusters 7, 8 and 10 reflected noticeable differences in NAAD^+^ correlations. In fact, higher levels of *CACYBP, CSNK1A1, CSNK1E, CSNK2B, DAAM1, MYC, PPP2CB, PPP2R5C, SMAD4, SIAH1*, and *WNT5B* expression were detected in S-231; however, these genes also revealed a stronger relationship with NAAD^+^ in S-231 than they did in P-231. Based on the gene-annotated Wnt signaling pathway and including all differentially expressed genes between the groups (*p* < 0.05), several genes indicated an enhancement of the Wnt signaling pathway and a special relationship with the Ca^2+^ signaling pathway (Figure S7). This suggests that S-231 exhibit genetic reprogramming in the Ca^2+^ signaling pathway and in nicotinate and nicotinamide metabolism, as well as in the Wnt signaling pathway. These findings indicate that cancer cells have adapted to glucose-deprived conditions and can modulate gene expression programs to convert to a CSCs state. One of the critical consequences of such environmental selective pressure is activation of Wnt signaling pathway leading to metabolic reprogramming to ensure survival in nutrient-deficient tumor microenvironment.

### NAAD^+^ levels are regulated by enzymes downstream of Wnt signaling pathway

**Figure 4:**
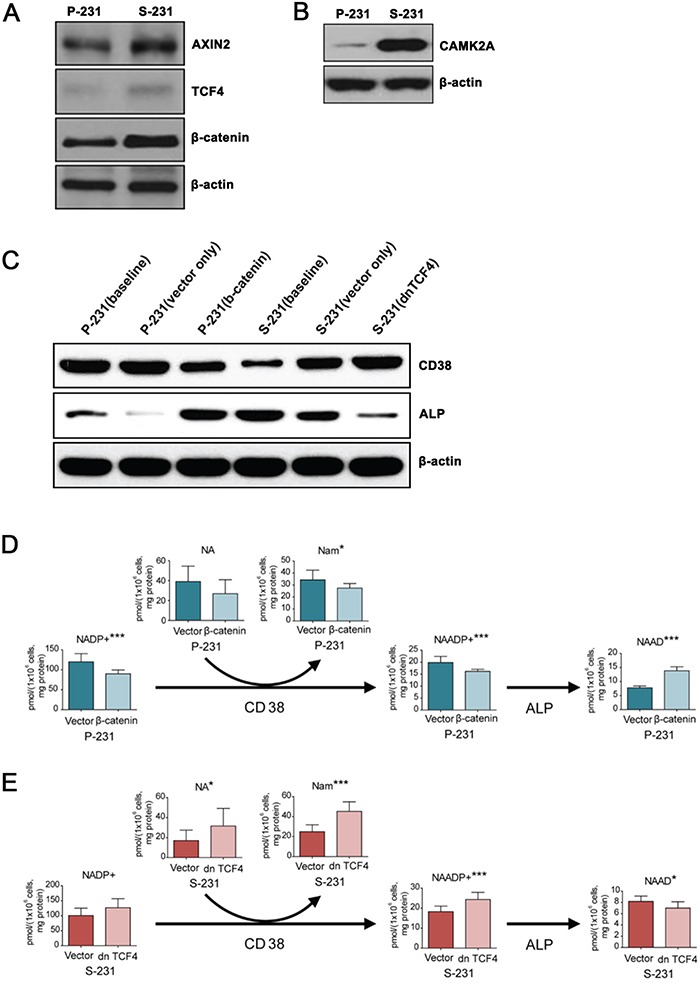
NAAD^+^ levels are regulated downstream of Wnt signaling by CD38 and alkaline phosphatase **A.** and **B.** Western blotting for canonical Wnt/β-catenin and non-canonical Wnt/Ca^2+^ signaling genes in P- and S-231. β-actin was used as a loading control. **C.** Protein levels of ALP and CD38 in β-catenin expressing P-231 and dnTCF4 expressing S-231. **D.** and **E.** Targeted metabolite analysis reveals changes in concentrations of NAAD^+^ derivatives in parental cells treated with either dsRed or β-catenin and S-231 treated with either dsRed or dn TCF4. Concentration is expressed in pmol per 1 × 10^6^ cells and corrected by protein (mg). Data are expressed as means ± standard deviation (S.D.). P-231, n = 10; S-231, n = 10. *, ** and *** indicate *p* < 0.05, < 0.01 and < 0.001, respectively. NAAD^+^, nicotinic acid adenine dinucleotide. NAADP, nicotinic acid adenine dinucleotide phosphate; NADP^+^, nicotinamide adenine dinucleotide phosphate (oxidized from); Nam, nicotinamide; NA, nicotinic acid.

To evaluate the functional relevance of the transcriptome results, we measured protein expression levels by western blot. In S-231, the expressions of Axin2, TCF4, and β-catenin, which are Wnt canonical pathway targets as well as that of calcium/calmodulin-dependent protein kinase II alpha (CAMK2A), a core Ca^2+^ signaling pathway protein involved in non-canonical Wnt signaling, were significantly higher than their levels in P-231 (Figure 4A and 4B).

Nicotinic acid adenine dinucleotide phosphate (NAADP) is a ubiquitous second messenger that provides a Ca^2+^ trigger in a wide range of cell types [23]. NAADP was shown to be degraded to NAAD^+^ by alkaline phosphatase (ALP) [24] and generated by the multifunctional enzyme CD38 [25]. It has been reported that the ALP expression induced by bone morphogenetic protein 2 (BMP2) and sonic hedgehog (SHH) is reliant on Wnt signaling [26]. To investigate the role of Wnt activity in ALP and CD38 expression in P- and S-231, TCF4 and β-catenin, two mission-critical transcription factors that mediate Wnt activity, were transfected with the active form of β-catenin into P-231, and a dominant negative form of TCF4 (dnTCF4) was transfected into S-231, respectively. We assessed the levels of ALP and CD38 proteins in P-231 and S-231 by western blot analysis and observed increased expression of ALP and decreased expression of CD38 in S-231 compared with P-231 at baseline (Figure 4C). When the active form of β-catenin was overexpressed in P-231, which mimics up-regulated Wnt signaling, ALP expression increased similar to the levels observed in S-231 (Figure 4C). In contrast, expression of dnTCF4 in S-231, which mimics down-regulated Wnt signaling, resulted in decreased relative expression of ALP as seen in P-231 (Figure 4C). We also found that CD38 expression levels were oppositely regulated by Wnt activity in P-231 and S-231 (Figure 4C).

Next, we wanted to investigate whether the level of NAAD^+^ is regulated by Wnt signaling. To this end, concentration changes in metabolites related to NAAD^+^ were evaluated in β-catenin overexpressing P-231 and dnTCF4 expressing S-231 as well as in vector control cells using LC-MS/MS. An increase in NAAD^+^ was observed in β-catenin overexpressing P-231, but all other metabolites in the pathway were downregulated (Figure 4D). On the other hand, the majority of NAAD^+^-related metabolites were upregulated in S-231 expressing dnTCF4, whereas NAAD^+^ itself was downregulated (Figure 4E). Furthermore, NAAD^+^ levels significantly increased when ALP was highly expressed, but decreased in the presence of low ALP expression. Therefore, these results suggest that the intracellular concentration of NAAD^+^ is correlated with that of ALP, the expression of which is regulated by Wnt signaling, and that NAAD^+^ accumulation is a consequence of the breakdown of NAADP as well as of metabolic changes in NAAD^+^ derivatives.

### NAAD^+^ levels are related to cytosolic Ca^2+^ levels and apoptotic cell death

**Figure 5:**
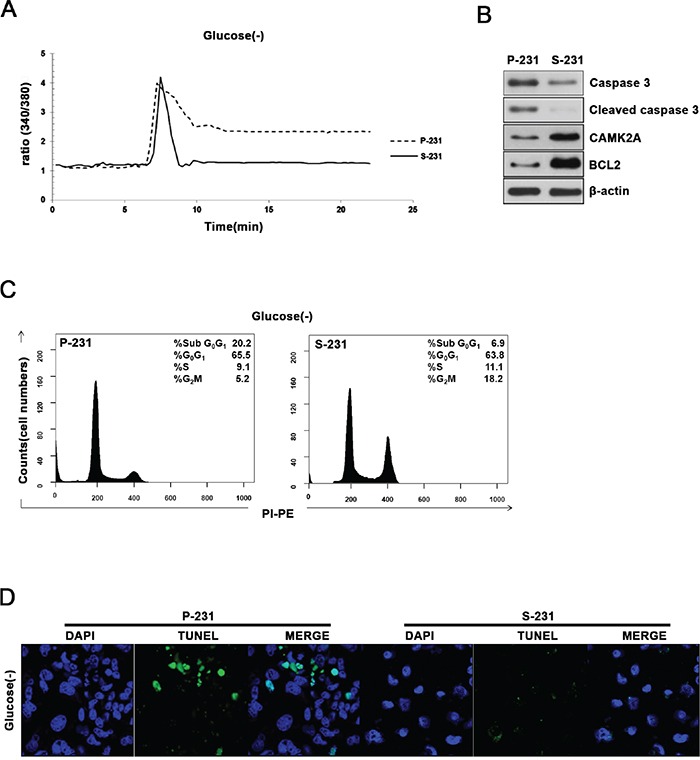
Elevated NAAD^+^ levels are related to restoration of cytosolic Ca^2+^ levels and escaped cell death **A.** Cytoplasmic free Ca^2+^ levels were measured in P-231 and S-231 on glucose-deprived conditions (40hrs) using the free Ca^2+^ indicator, Fura-2AM. **B.** Immunoblot analysis for apoptosis markers caspase 3 and BCL2 as well as CAMK2A in P-231 and S-231 upon glucose-deprived condition. **C.** Flow cytometry analysis of P-231 and S-231 in glucose-deprived conditions **D.** Detection of apoptosis by TUNEL assay. Green fluorescence TUNEL-positive cells were noted in P-231. Scale Bar, 20 μm.

To investigate these alterations in signaling and metabolic pathways could provide selective advantage to CSCs, we examined the cell viability and cytosolic Ca^2+^ levels since glucose deprivation causes an elevation of intracellular Ca^2+^ that subsequently triggers apoptosis when persistently remain elevated. Indeed, the cytosolic Ca^2+^ levels failed to return to the basal levels in P-231 while returned to the basal levels in S-231 after the spike of intracellular Ca^2+^ in the absence of glucose (Figure 5A). Western blot analysis demonstrated that S-231 exhibited increased expression of anti-apoptotic BCL2 and CAMK2A while decreased expression of cleaved caspase 3 indicating that apoptosis was inhibited in glucose deprivation when compared with P-231 (Figure 5B). To further elucidate whether decreased cytosolic Ca^2+^ concentration in glucose deprivation leads to anti-apoptosis, we analyzed proportion of cell cycles using FACS and performed TUNEL assays to visualize apoptotic cells. Indeed, cell cycle analysis indicated that the fraction of sub G0/G1 cells of P-231 was greater than that of S-231 (Figure 5C). A TUNEL assay also revealed that more DNA fragmentation in P-231 than in S-231 (Figure 5D). Together, these results suggested that elevated NAAD^+^ levels might be related to cytosolic Ca^2+^ level restoration to the basal state, providing protection of the CSCs from glucose-deprived cell death.

## DISCUSSION

In this study, we investigated metabolic stress-resistant stem-like cancer cells that were phenotypically converted and had acquired cancer stem-ness properties by the adaptive response to gradual nutrient depletion in an *in vitro* system [14]. These stem-like cancer cells were compared to unmodified cancer cells by metabolic and transcriptional profiling. Metabolic profiling found that S-231 cells exhibited specific metabolic changes including alterations of calcium and NAD^+^ metabolism in particular. Network analysis of transcriptome profiling showed that activated Wnt signaling is a key pathway for developing stem-like cancer cell state. The linking density of the Wnt network was found to be more dramatically altered in S-231 than in P-231 regardless of whether the culture contained glucose or not. Our integrated analysis of the cellular metabolome with transcriptome profiling (Figure S8) identified the Wnt pathway mediated metabolic reprogramming as catalyzed by the regulatory enzyme ALP. In NAD^+^ metabolic network, differentially expressed genes in S-231 might cause an accumulation of NAAD^+^ metabolites. NAAD^+^ can be synthesized either from nicotinic acid (NA) through de novo biosynthesis or from catalytic conversion through dephosphorylation of NAADP. Of note, the genes differentially expressed between P-231 and S-231 showed that a key biosynthetic enzyme, i.e. NMNAT2, among others was not increased in S-231; rather it was downregulated (Table S3). Thus, we assume that the increased level of NAAD^+^ in S-231 is not due to de novo biosynthesis but due to bioconversion reaction from NAADP catalyzed by ALP that is upregulated through Wnt signaling pathway. Together, these results suggest that Wnt signaling activity in CSCs instigates reprograming of the metabolic-subnetwork thereby resulting in alterations in NAD^+^ metabolites.

In Table S3, among most significantly differentially represented genes were *CTNNBIP1* and *WNT5B* involved in Wnt/−Ca^2+^ signaling pathways. WNT5B is able to activate calcium signaling and expression of WNT5B was increased in advanced stage gastric cancer and correlated with poor prognosis [29, 30]. *CTNNBIP1* of which expression is decreased in S-231 is a negative regulator of the Wnt signaling pathway, preventing β-catenin from interacting with TCF. Together, transcriptional reprogramming in S-231 leads to activation of Wnt and Wnt-mediated Ca^2+^ signaling pathway thereby promoting stem-like properties and cell survival in metabolic stress environment.

Wnt-Ca^2+^ signaling leads to transient increases in cytoplasmic free calcium that subsequently activate the kinases protein kinase C (PKC), CAMK2A and protein phosphatase 3, calcineurin [27, 28]. ALP is a downstream enzyme in the Wnt signaling pathway and its expression is modulated by Wnt activity [26, 29]. In contrast, the relationship between CD38 and the Wnt pathway is not clearly established. CD38, also known as cyclic ADP ribose hydrolase, is found on the surface of many cell types, and functions in diverse biological processes including signal transduction and calcium signaling [30]. CD38 has been identified as a poor prognostic factor for chronic lymphocytic leukemia. Despite these, the potential role of CD38 in neoplastic transformation and tumor progression remains elusive [31], as no direct regulatory mechanisms have been elucidated. Wnt, of note, has been reported to be important for increasing the CD34^+^/CD38^−^ hematopoietic stem cell populations [32]. Taken together, these results strongly suggest that Wnt pathway in stem-like cancer cells regulates the expression of both CD38 and ALP, thereby fine-tuning the levels of NAADP and NAAD^+^ according to the context-dependent cellular needs.

In metabolically challenging conditions, maintenance of cell viability is largely dependent on glucose deprivation which induced the elevation of cytosolic free Ca^2+^ by endoplasmic reticulum stress [33, 34]. In cells, the endoplasmic reticulum is the major Ca^2+^ storage site, although mitochondria also play an important role in Ca^2+^ homeostasis. Mitochondria can rapidly uptake cytosolic Ca^2+^, acting as a buffer when the level of cytosolic free Ca^2+^ is increased. Notably, a subsequent increase in Ca^2+^ in the mitochondria temporarily augments respiration, thereby compensating cellular bioenergetics homeostasis during acute glucose starvation [35–37]. However, Ca^2+^ overload is deleterious, as it decreases mitochondrial respiration, leading to a decline in membrane potential, mitochondrial swelling, cytochrome c release, and finally the triggering of apoptotic cell death [38, 39]. Thus, it is obvious that an active lowering of cytosolic Ca^2+^ levels is critical for the ability of cancer cells to survive glucose deprivation-induced apoptosis.

In summary, the results show that the selective increase in NAAD^+^ as a consequence of the cross-talk between metabolic network and deranged cancer signaling pathway provides a survival advantage to stem-like cancer cells. Mechanistically, Wnt-dependent upregulation of ALP is accountable for degrading NAADP, a Ca^2+^ mobilizing messenger which increases free cytosolic Ca^2+^, to NAAD^+^, reversing the cytosolic Ca^2+^ levels thereby protecting cells from apoptosis.

## MATERIALS AND METHODS

### Cell culture and generation of selected cancer stem-like cell lines

Human breast cancer cell lines MDA-MB-231 (parental) cells and chronic metabolic stress (CMS)-selected MDA-MB-231 cells from MDA-MB-231 parental cells were cultured in RPMI-1640 with 5% FBS in a humidified incubator containing 5% CO_2_ at 37°C. MDA-MB-231 cells were cultured without refreshment of the culture medium and recovered for several rounds. Briefly, cancer cells were initially seeded in standard culture medium and continued in culture without medium change until 90% of the cancer cells had died. This process was designated as *in vitro* CMS culture. The remaining viable cells were collected and subjected to another round of CMS culture conditions for several rounds

### Constructs and transfections

Transfections into S-231 were carried out using 1.6 μg pPGS-neo or 80 μg pPGS-dnTCF4 in 100 μL Opti-MEM (Gibco). At the same time, 4 μL Lipofectamine 2000 was added to 100 μL Opti-MEM. After 5 min incubation, the mixtures were combined, mixed gently, and incubated for 25 min at room temperature. The growth medium (1 mL) was removed from the cells, and the 200 μL complexes were added to each well. After 4-6 hours, the medium was changed. Subsequent selection of the bulk cell population in G418 was performed at an initial concentration of 1 mg/mL. After 48 h, the G418 concentration was reduced to 0.75 mg/mL. After 1 week, the G418 concentration was further reduced to 250 μg/mL and the expression of the transferred genes was confirmed.

### Western blot analysis

Cells were extracted in RIPA buffer (50 mM Tris-HCl (pH 7.5), 150 mM NaCl, 1 mM Na2EDTA, 2 mM EGTA, 1% triton X-100, 1% sodium deoxycholate, 2.5 mM sodium pyrophosphate), PMSF and protease inhibitor (Roche, 11873580001). Equal amounts of protein, which was determined by BCA assay, were electrophoresed in 10% SDS-PAGE gels, transferred to PVDF membrane (Bio-Rad). Membranes were blocked with 5% skim milk, and primary antibody incubations were performed at 4°C for overnight caspase-3 (Santa Cruz, CA, USA), Axin2 (Santa Cruz), TCF4 (Santa Cruz), BCL2 (Santa Cruz), β-catenin (Abcam, Cambridge, UK), CAMK2A (Abcam), β-actin (Sigma) 1:1,000 dilution). And the membranes were incubated with HRP-conjugated secondary antibody (1:5,000) and detected by chemiluminescense with Supersignal West Pico or Femto substrate (Thermo Scientific) on AGFA medical X-ray film blue.

### Sphere formation assay

To generate mammospheres, 1×10^5^ cells/mL were seeded in StemFit 3D cell culture dish (Microfit, Seoul, Korea). This dish with concave micromolds of 500 μm diameter were fabricated using soft lithography techniques. The concave microwells were coated with 3% (w/v) bovine serum albumin (BSA) overnight to prevent cell attachment. After 2day, the number of mono-cultured spheroids evaluated.

### Flow cytometry cell cycle analysis

Cells were treated with glucose-deprived RPMI 1640 medium with 10% FBS for 40 hours, harvested by trypsinization, and fixed with 70% ethanol. Cells were stained for total DNA with a solution containing 40 μg/ml propidium iodide (PI) and 100 μg/ml RNase I in PBS for 30 min at 37°C. Cell cycle distribution was then analyzed with the FACS Calibur Flow Cytometer (BD Biosciences, SanJose, CA, USA). The proportions of cells in the G0/G1, S, and G2/M phases were analyzed by FACS and DNA software program (FlowJo v8, MacOSX, Tree Star, Ashland, OR, USA). This experiment was repeated three times, and the results were averaged.

### Cytosolic calcium measurement

Cells were perfused with 140 mM NaCl, 5.4 mM KCl, 2 mM CaCl_2_, 1 mM MgCl_2_, 33 mM glucose, and 20 mM HEPES (pH 7.4, adjusted with NaOH, and 320–350 mOsm with sucrose). The intracellular Ca^2+^ level of MDA-MB-231 cells was imaged using a calcium-sensitive fluorescent dye, Fura-2AM. Fluorescence intensities (ΔF) were normalized to the resting values.

### TUNEL assays

For the measurement of apoptotic cells, cells were fixed with 4% paraformaldehyde solution for 48 hours and stained using a Terminal Deoxynucleotidyl Transferase dUTP Nick End Labeling (TUNEL) kit (Promega, Madison, WI, USA). The apoptotic cells (fluorescent green) and total cells were counted under a fluorescence microscope, and the data were recorded. Images were collected using a confocal microscope (LSM Meta 700, Carl Zeiss, Oberkochen, Germany) and were analyzed using the Zeiss LSM Image Browser software program, version 4.2.0121.

### Microarray experiment and data analysis

Total RNA was isolated from cells harvested after each treatment using the mirVanaTM miRNA Isolation Kit (Ambion) according to the manufacturer's protocol. Biotin labeled cRNA was prepared using the Illumina TotalPrep RNA Amplification Kit (Ambion). A 500 ng aliquot of total RNA was used for the synthesis of cDNA followed by amplification and biotin labeling as recommended by the manufacturer. From this, 1.5 μg biotinylated cRNA per sample was hybridized to an Illumina Human-6 BeadChip v.2 microarray and signals were developed by Amersham fluorolink streptavidin-Cy3 (GE Healthcare Bio-Sciences). Data were analyzed using Illumina Bead Studio v3.0 after scanning with an Illumina bead Array Reader confocal scanner (BeadStation 500GXDW; Illumina Inc.). All statistical analyses were performed using R 2.3.0 and BRB Arraytools Version 3.5 (http://linus.nci.nih.gov./BRB-ArrayTools.html) with quantile normalization.

To minimize the effect of variation from non-biological factors, the values of each sample were normalized using a quantile normalization method. A random-variance *t*-test was applied for the calculation of significance of each gene in the comparison of two classes and one-way ANOVA was applied for the evaluation of significance in multi group comparison. Cluster analysis was performed with Cluster and Treeview (http://rana.lbl.gov/EisenSoftware.htm). For cluster analysis, log base 2 transformed data were centered to the mean values of each gene's expression. GSEA (below) was performed against GO of biological processes and the Kolmogorov-Smirnov statistic was applied for the evaluation of the statistical significance of each GO category.

### GSEA

GSEA is a computational method that determines whether an a priori defined set of genes shows statistically significant, concordant differences between two biological states [40, 41].

### Wnt network analysis

We collected member genes of the Wnt pathway from the KEGG database. We computed the Pearson's correlation between pairs of member genes of Wnt pathways to generate Wnt co-expression values for both P-231 and S-231 cells. Edges represented correlations between two member genes of the Wnt pathway in each condition. We removed negative correlations and only considered positive correlations in our analysis.

To detect a set of genes that was differentially co-expressed across two conditions (such as genes that were strongly co-expressed in selected but not in parental cells), we computed and selected edges that were different between two Wnt co-expression networks (for example, those with differences of edge values > 0.6) between selected and parental cell Wnt co-expression networks. Selected edges indicated that the two genes linked with the selected edge were strongly co-expressed in the S-231 cells but not in the parental cells.

Finally, we generated network views of selected Wnt co-expression networks that were strongly co-expressed in S-231.

### Sample preparation for metabolic analysis

MDA-MB 231 cells (2 × 10^7^) were quenched with cold methanol to stop cellular metabolism. For the extraction of intracellular metabolites, freeze-thaw cycles with 80% aqueous methanol were used as described previously [42]. Cell extracts were collected, dried under vacuum and resuspended in deuterium oxide solution buffered with 100 mM aqueous sodium phosphate (300 μL, pH 7.0 ± 0.01), vortexed for 30 s, and centrifuged at 16,600 × *g* for 1 min at 4°C. The supernatant (266 μL) was transferred into the 5-mm outer tube of an NMR 517 coaxial insert (two tubes). Next, the inner tube containing 0.5 mM 3-(trimethylsilyl)-1-propanesulfonic acid-d_6_ sodium salt (DSS-d_6_, 98 atom %) was inserted into the outer tube.

### NMR measurements

Samples were analyzed using a VNMRS 600-MHz NMR spectrometer equipped with a triple-resonance, HCN salt-tolerant cold probe (Agilent Technologies Inc.). A NOESYPRESAT (RD–90°–t1–90°–tm–90°–FID acquisition) pulse sequence was applied to suppress the residual water signal at 25°C. We collected 128 transients into 67,568 data points using a spectral width of 8445.9 Hz with a relaxation delay of 2.0 s, an acquisition time of 4.00 s, and a mixing time of 100 ms. A 0.5 Hz line-broadening function was applied to all spectra prior to Fourier transformation.

### Data processing and multivariate analysis

All acquired FIDs were Fourier transformed, phase corrected and aligned to the chemical shift of DSS-d_6_ at 0 ppm using Varian software VNMRJ version 2.2C (Agilent Technologies). The ^1^H NMR spectra of cell extracts were then baseline-corrected using the Processor module of the Chenomx NMR suite version 7.1 (Chenomx Inc.). Assignments of NMR signals were based on total correlation spectroscopy (2D ^1^H-^1^H TOCSY; Figure S9), spiking experiments, and comparison to previous reports. The identification of metabolites and determination of concentration were performed using the Profiler module of Chenomx NMR suite version 7.1. The data were then normalized to the total protein concentration, as determined by the Bradford assay. The resulting ^1^H NMR data were imported into SIMCA-P version 12.0 (Umetrics) for chemometric analyses. All imported data sets were mean-centered and scaled to unit-variance, which gives base weight to the data sets. Subsequently, supervised regression analysis was conducted using PLS-DA to examine the differences among groups. The fit of the model to the data was described by *R^2^* and *Q^2^* values (where *R^2^* describes the goodness of fit and *Q^2^* indicates predictability). A VIP column plot was generated to identify the metabolites responsible for the differences among groups.

### Solid-phase extraction (SPE) and sample preparation

Cells (2 × 10^6^) extracted by the same method described for NMR spectroscopy were purified by solid-phase extraction to enhance the sensitivity for NAAD^+^-related metabolites. The extracts were applied to a C_18_ cartridge (Oasis HLB^®^, Waters) preconditioned with 3 mL methanol followed by 3 mL H_2_O. The sample was eluted with 2 mL H_2_O and 2 mL H_2_O-methanol (30:70, v/v). The elute was then dried under vacuum, resolved in 200 μL H_2_O-acetonitile (50:50, v/v) containing 10 mM ammonium acetate adjusted to pH 9.0 with ammonium hydroxide and filtered with 0.22-μm PTTE (Merck Millipore). An internal standard (nicotinic acid, ethyl ester-D4) was added to the sample at a final concentration of 250 ng/mL.

### LC-MS/MS measurements

LC-MS/MS analysis of NAAD^+^-related metabolites was performed using an Agilent 1290 Infinity LC and Agilent 6490 Triple Quadrupole MS system equipped with an Agilent Jet Stream ESI source (Agilent Technologies). MassHunter Workstation (ver. B.06.00, Agilent Technologies) software was used for data acquisition and analysis. Chromatographic separation was carried out on an ethylene-bridged hybrid (BEH) Amide column (100 × 2.1 mm with a 1.7-μm particle size, Waters) at a flow rate of 0.2 mL/min. The column and auto-sampler were maintained at 25°C and 4°C, respectively. For each run, a sample volume of 2 μL was injected, and the total run time was 9 min. Mobile phases A and B were 10 mM ammonium acetate in H_2_O and H_2_O-acetonitile (10:90, v/v) adjusted to pH 9.0 with ammonium hydroxide, respectively. The gradient elution began with 50% mobile phase A for 3 min, increased to 95% mobile phase A for 4 min, and was held for 1 min. The composition was then returned to the initial conditions at 5.1 min and maintained for 3.9 min for equilibration purposes. Data were acquired by single reaction monitoring in positive-ion mode. Optimized parameters for each compound were obtained using a flow injection of 100 ng/mL individual standard compounds resolving H_2_O-acetonitile (50:50, v/v) containing 10 mM ammonium acetate (pH 9.0) into MS. The performance parameters for LC-MS/MS used for the quantification of compounds are described in Table S4. MS/MS experiments were conducted with the following parameters: capillary voltage, 2.5 kV; nebulizer gas of nitrogen, 40 psi; drying gas temperature, 180°C; drying gas flow rate, 11 L/min; sheath gas temperature, 370°C; sheath gas flow rate, and 12 L/min; nozzle voltage, 0 V.

### Correlation analysis for integrated transcriptomics and metabolomics

A total of 12,705 genes identified by microarray analysis were separated and categorized to investigate related pathways in the human KEGG pathway database. Genes with no significant detection levels were excluded. Genes involved in the Ca^2+^ signaling pathway, nicotinate and nicotinamide metabolism, and Wnt signaling were selected, and the Pearson's correlation coefficients with NAAD^+^ metabolites were calculated using Statistical Package for the Social Sciences (SPSS) software. Correlation matrices were illustrated with correlation coefficients ranging from −0.7 to +0.7 using Multiexperiment Viewer (MeV, version 4.9).

### Statistical analysis

Student's *t*-tests were performed using SPSS software to identify significant differences in metabolite levels. Differences were tested to a 95% probability level (*p* < 0.05).

## CONCLUSION

The integrated analysis of transcriptomic and metabolic profiling demonstrates an intricate survival mechanism of intertwined gene-metabolite modules that provides a selective advantage to CSCs. Altered NAD^+^ metabolism and, in particular, the elevation of NAAD^+^ which curbs cytosolic Ca^2+^ overload thereby preventing calcium-induced apoptosis in stress conditions might constitute a novel “oncometabolite” in CSC. Thus, it might represent a metabolic vulnerability in stem-like cancer cells that could in principle be targeted for therapeutic exploitation.

## SUPPLEMENTARY FIGURES AND TABLES




