# Supplementary material for: Integrated omics-analysis reveals Wnt-mediated NAD+ metabolic reprogramming in cancer stem-like cells
Source: Oncotarget. 2016 Jul 6;7(30):48562–76. doi: 10.18632/oncotarget.10432 (PMC5217038; doi:10.18632/oncotarget.10432)
Supplement: Supplementary file 1 [file oncotarget-07-48562-s001.pdf]

Integrated omics-analysis reveals Wnt-mediated NAD+ metabolic reprogramming in cancer stem-like cells

SUPPLEMENTARY FIGURES AND TABLES

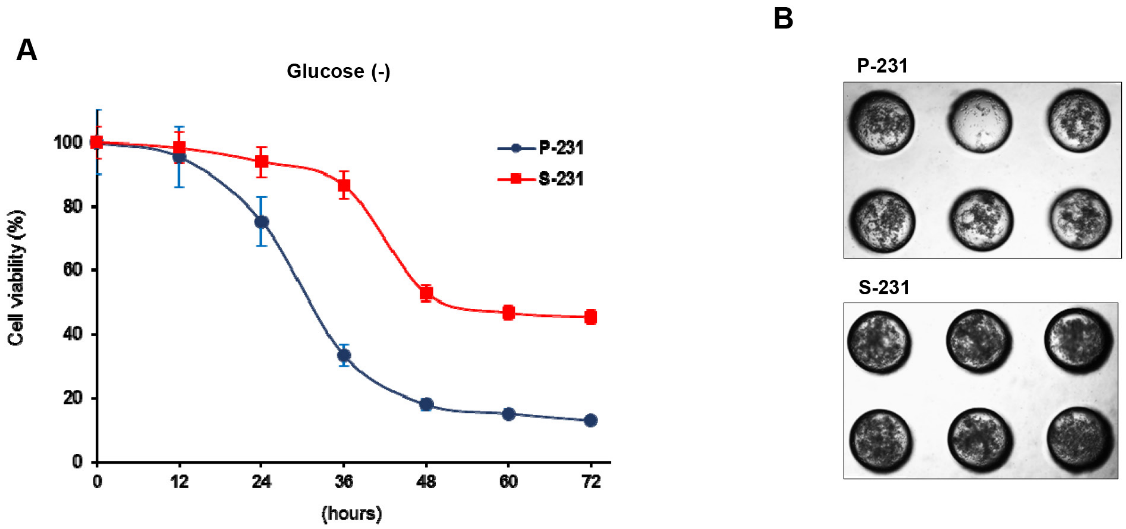

Supplementary Figure S1: Phenotype of stem-like cancer cells. A. Cell viability of P-231 and S-231 in glucose deprivation; **B.** Sphere formation of P-231 and S-231. Sphere forming ability shows to be one of characteristics of CSCs and promotes by reprogramming factors. The capacity of sphere formation is increased more S-231 than P-231.

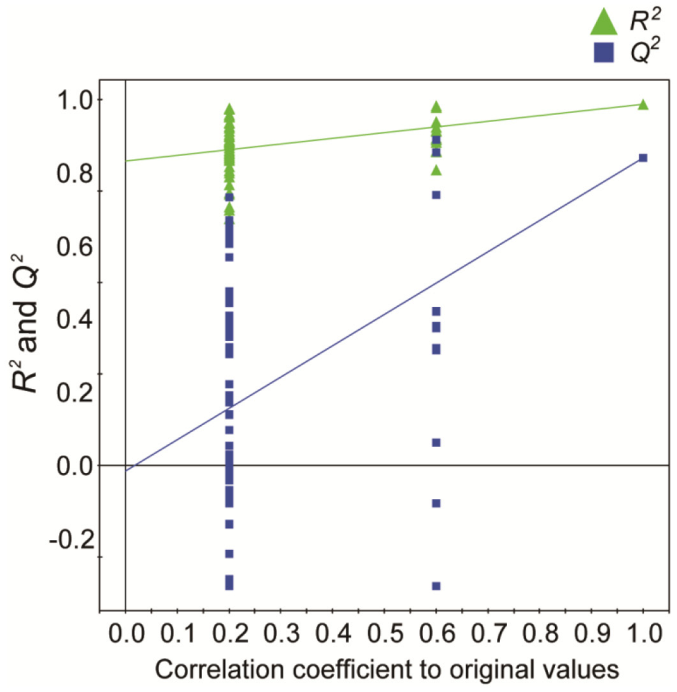

Supplementary Figure S2: Permutation plot demonstrating the validity of the PLS-DA model using a 100-permutation test of three components. Intercepts: R^2 = 0.834 (green triangles) and Q^2 = -0.056 (blue squares).

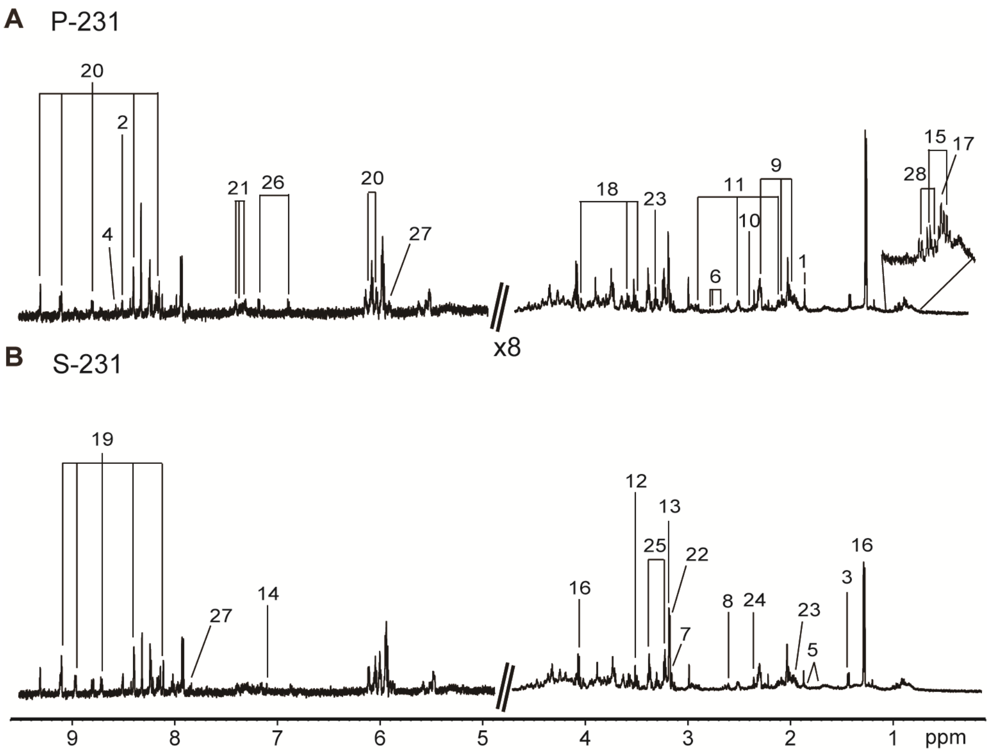

Supplementary Figure S3: Representative ^{1}H NMR spectra of parental and stem-like cancer cell extracts. A. P-231; **B.** S-231. 1, acetate; 2, adenosine diphosphate (ADP); 3, alanine; 4, adenosine monophosphate (AMP); 5, arginine; 6, aspartate; 7, choline; 8, citrate; 9, glutamate; 10, glutamine; 11, glutathione; 12, glycine; 13, glycero-3-phosphocholine (sn-GPC); 14, histidine; 15, isoleucine; 16, lactate; 17, leucine; 18, myo-inositol; 19, nicotinic acid adenine dipnucleotide ($NAAD^{+}$); 20, nicotinamide adenine dinucleotide (oxidized form, NAD^{+}); 21, phenylalanine; 22, O-phosphocholine (O-PC); 23, proline; 24, succinate; 25, taurine; 26, tyrosine; 27, uridine; 28, valine.

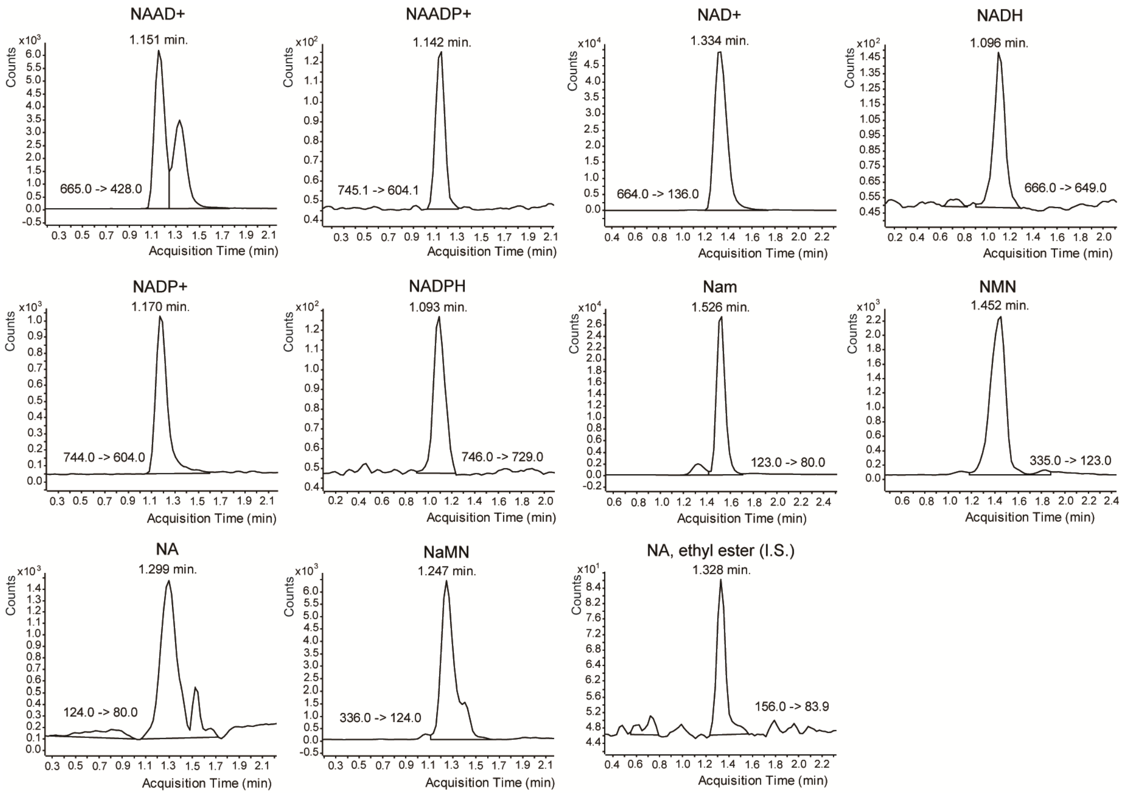

Supplementary Figure S4: Representative extracts ion chromatograms of NAAD$^+$ metabolites in cultured cell extracts. Abbreviations: NAAD$^+$, nicotinic acid adenine dinucleotide; NAADP$^+$, nicotinic acid adenine dinucleotide phosphate; NAD$^+$, nicotinamide adenine dinucleotide (oxidized form); NADH, nicotinamide adenine dinucleotide (reduced form); NADP$^+$, nicotinamide adenine dinucleotide phosphate (oxidized form); NADPH, nicotinamide adenine dinucleotide phosphate (reduced form); Nam, nicotinamide; NMN, nicotinamide mononucleotide; NA, nicotinic acid; NaMN, nicotinic acid mononucleotide.

A

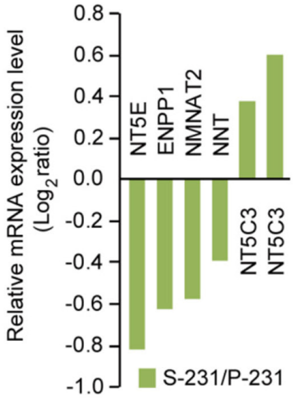

B

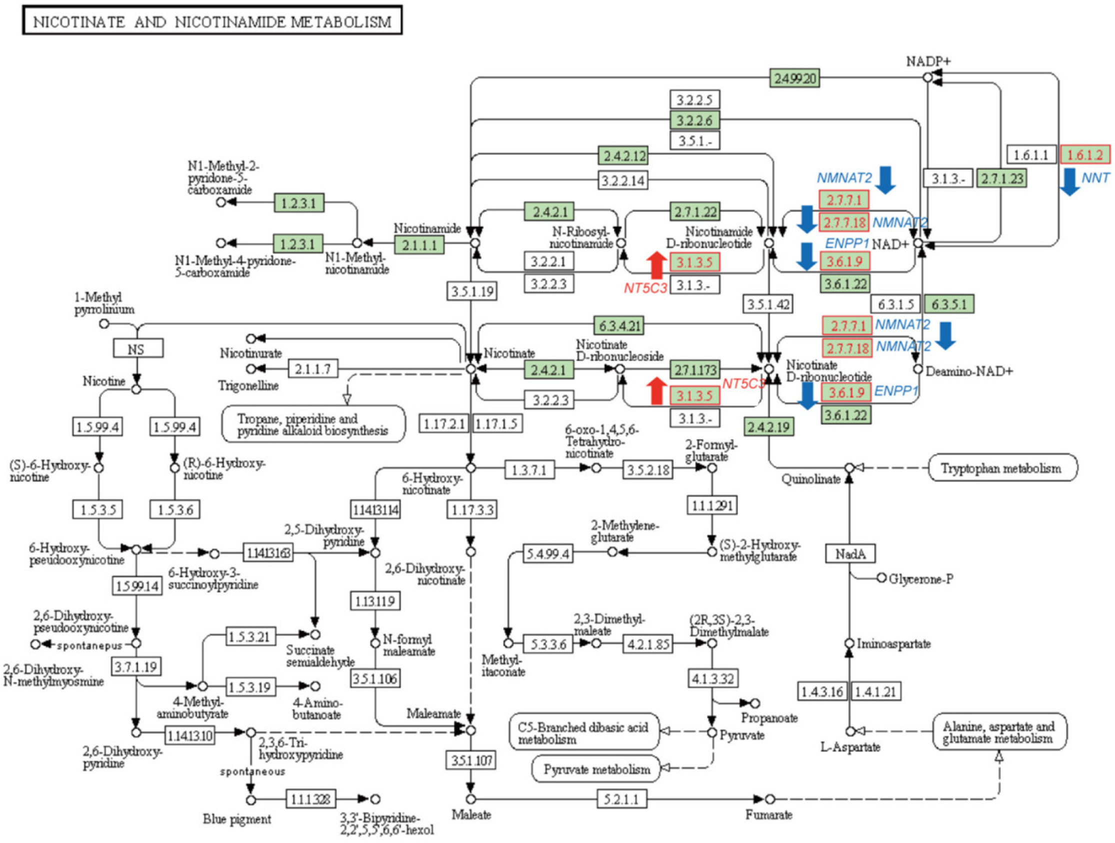

Supplementary Figure S5: Genetic variations in nicotinate and nicotinamide metabolism between P-231 and S-231. A. Differentially expressed genes in S-231 cells in nicotinate and nicotinamide metabolism ($p < 0.05$). Relative expression levels of mRNA are expressed with log2 ratio of intensities of S-231 versus P-231 and ordered according to levels of log2 ratio. **B.** Gene-annotated nicotinate and nicotinamide metabolism revealing expectations for decreased $NAAD^+$ production. Light green indicates endogenous genes in humans. Genes boxed in red indicate significant difference between P-231 and S-231 ($p < 0.05$). Letters and arrows colored red and blue represent up-regulation and down-regulation in S-231 cells compared to P-231 cells, respectively.

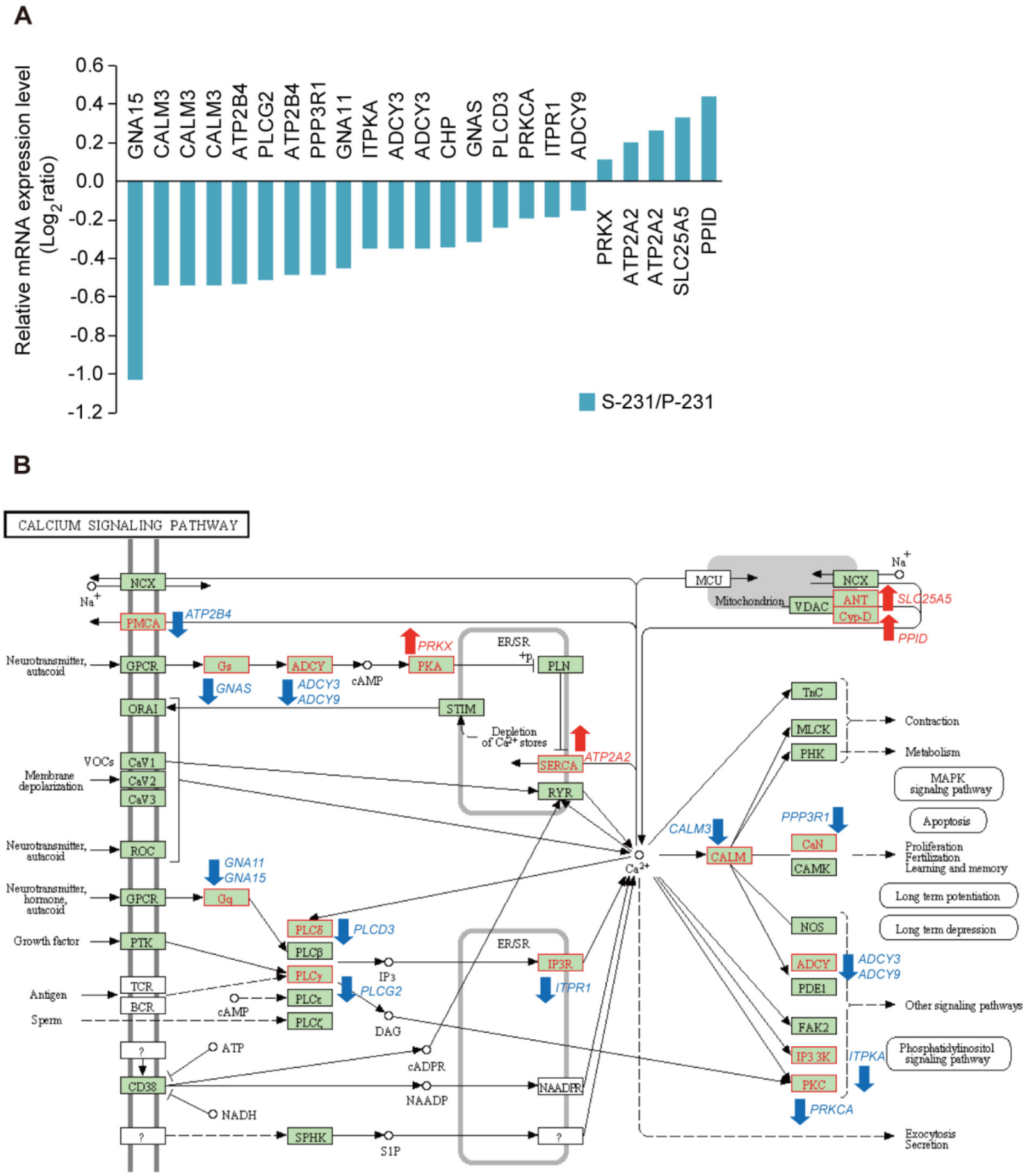

Supplementary Figure S6: Gene expression profile of S-231 revealing flow changes in free Ca^{2+} in cellular organelles. **A.** Differentially expressed genes in S-231 on Ca^{2+} signaling pathway ($p < 0.05$). Relative expression levels of mRNA are expressed with log2 ratio of intensities of S-231 versus P-231 cells and ordered according to levels of log2 ratio. **B.** Gene-annotated Ca^{2+} signaling pathway indicating reduction of Ca^{2+}secretion to cytoplasm and increase of Ca^{2+} accumulation into endoplasmic reticulum and mitochondrial excretion. Light green means endogenous genes in human. Genes boxed in red indicate significant difference between P-231 and S-231 ($p < 0.05$). Letters and arrows colored red and blue represent up-regulation and down-regulation in S-231 compared to P-231, respectively.

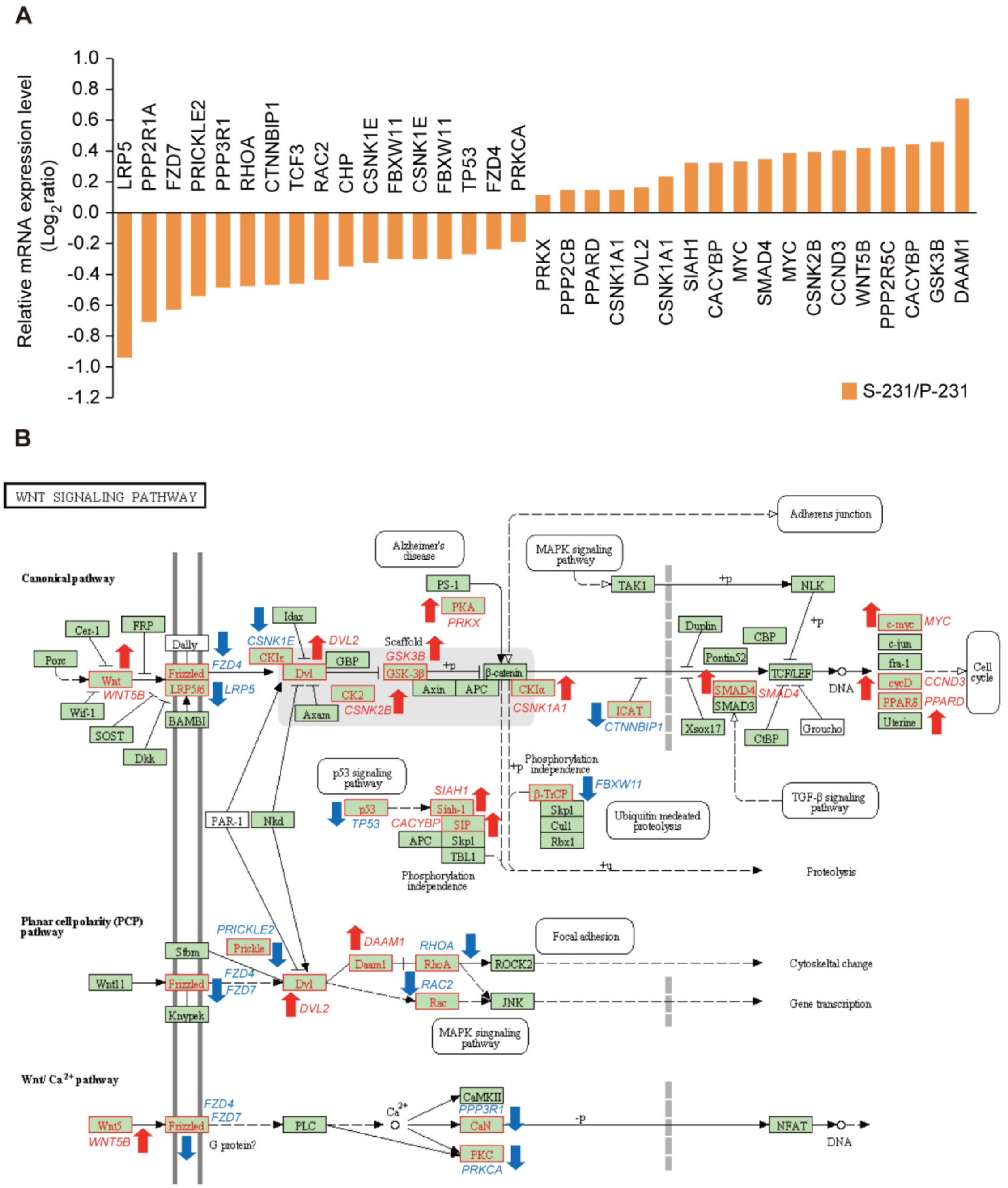

Supplementary Figure S7: Differentially expressed genes related to enrichment of the Wnt signaling pathway in stem-like cancer cells. A. Gene expression levels in S-231 in Wnt signaling pathway ($p < 0.05$). Relative expression levels of mRNA are expressed with log2 ratio of intensities of S-231 versus P-231 and ordered according to levels of log2 ratio. **B.** Gene mapping pathway indicating enhancement of Wnt signaling pathway and special connection to Ca^{2+} signaling pathway. Light green indicates endogenous genes in humans. Genes boxed in red indicate significant difference between P-231 and S-231 ($p < 0.05$). Letters and arrows colored red and blue represent up-regulation and down-regulation in S-231 compared to P-231, respectively.

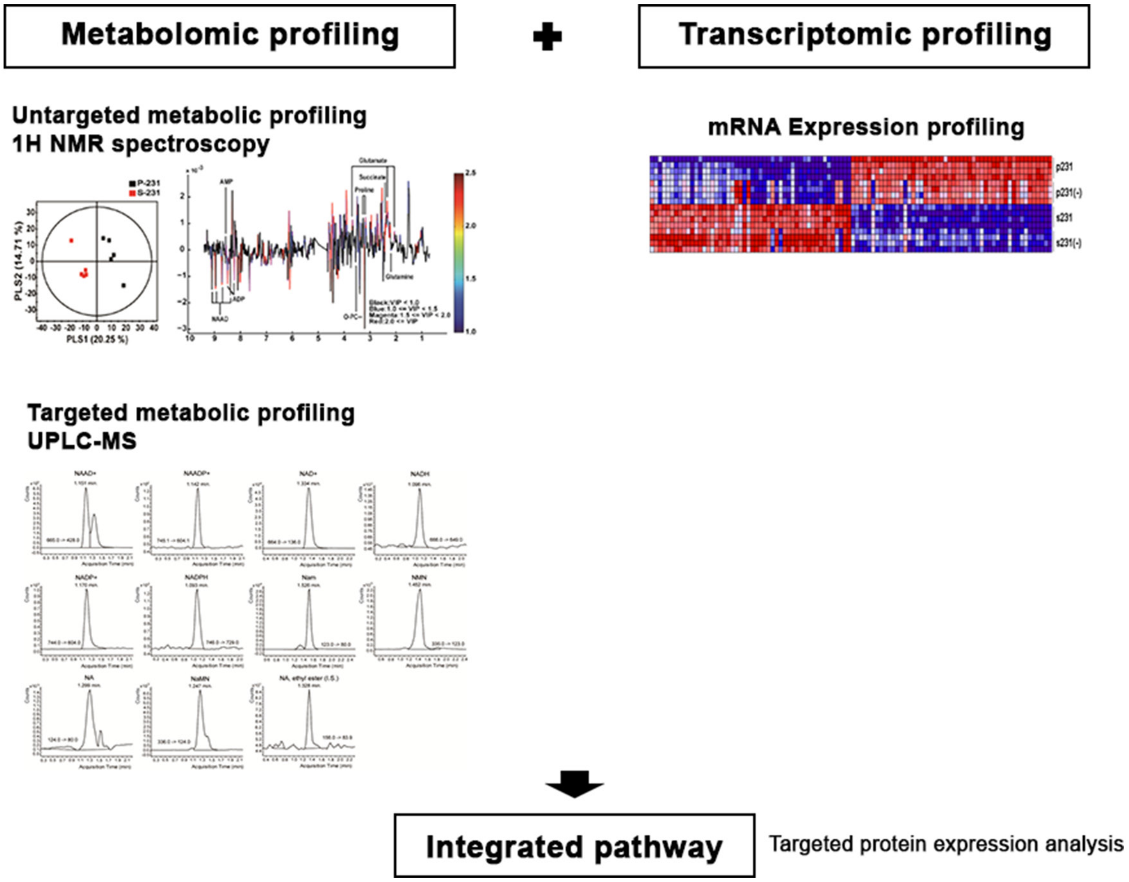

Supplementary Figure S8: Overview of Integrated metabolome and transcriptome approaches.

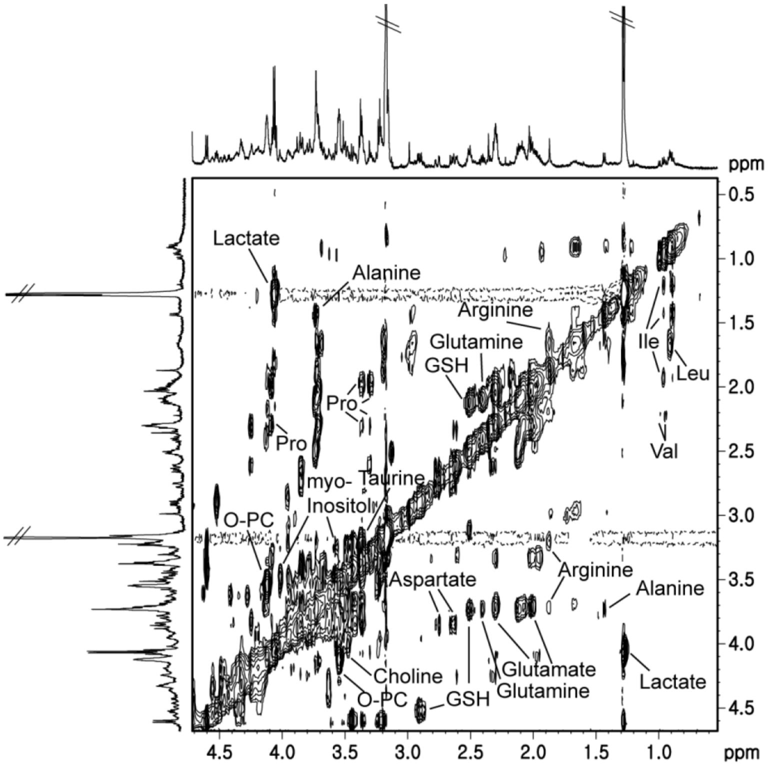

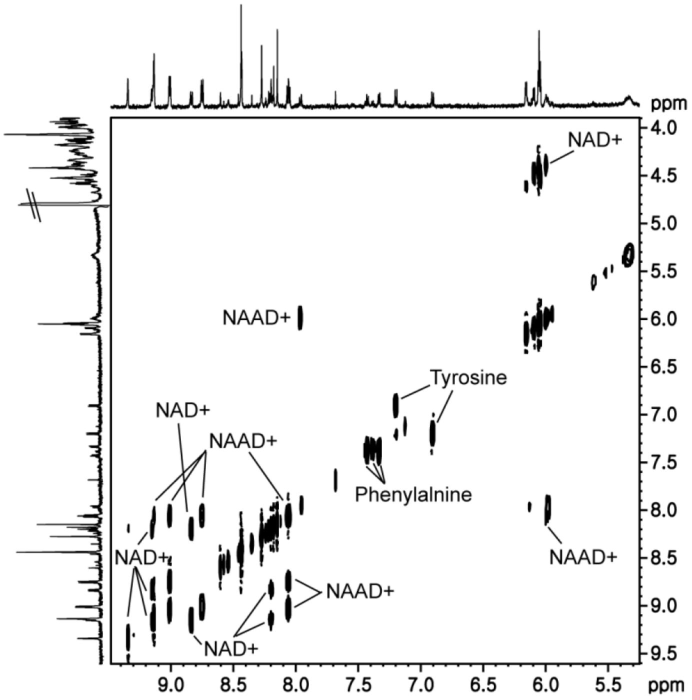

Supplementary Figure S9: Representative TOCSY spectra of stem-like cancer cell extracts. Abbreviations: GSH, glutathione; Ile, isoleucine; Leu, leucine; NAAD$^+$, nicotinic acid adenine dinucleotide; NAD$^+$, nicotinamide adenine dinucleotide (oxidized form); O-PC, O-phosphocholine; Pro, proline; Val, valine.

Supplementary Table S1: Intracellular metabolites, their chemical shifts, and fold changes from ^{1}H NMR spectra in parental and selected cells

See Supplementary File: 1

Supplementary Table S2: Intracellular metabolites and fold changes in parental and selected cells detected by LC-MS/MS

ID	(+) P-231	(+) S-231	(-) P-231	(-) S-231	Fold change		p-value[a]	
					(+) S/(+) P	(-) S/(-) P	(+) S/(+) P	(-) S/(-) P
NAAD+	17.48 ± 2.35	46.24 ± 6.14	6.95 ± 0.56	11.02 ± 0.77	2.645	1.586	< 0.001	< 0.001
NAADP+	1.74 ± 0.24	2.33 ± 0.42	1.35 ± 0.12	1.68 ± 0.23	1.337	1.244	0.028	0.020
NAD+	45.23 ± 4.57	42.14 ± 4.59	72.88 ± 8.77	71.82 ± 2.48	0.932	0.985	0.317	0.802
NADH	0.13 ± 0.02	0.16 ± 0.03	0.12 ± 0.02	0.17 ± 0.11	1.215	1.405	0.134	0.354
NADP+	1.08 ± 0.18	1.47 ± 0.29	1.07 ± 0.14	1.45 ± 0.22	1.355	1.356	0.036	0.012
NADPH	0.1 ± 0.02	0.16 ± 0.04	0.13 ± 0.02	0.23 ± 0.2	1.619	1.756	0.014	0.295
Nam	0.54 ± 0.06	0.43 ± 0.02	0.36 ± 0.05	0.29 ± 0.04	0.791	0.808	0.003	0.046
NMN	0.54 ± 0.06	0.63 ± 0.16	0.06 ± 0.02	0.07 ± 0.02	1.176	1.243	0.254	0.244
NA	0.09 ± 0.03	0.16 ± 0.03	0.06 ± 0.01	0.07 ± 0.02	1.906	1.18	0.002	0.355
NaMN	0.19 ± 0.05	0.43 ± 0.16	0.06 ± 0.02	0.09 ± 0.01	2.224	1.611	0.014	0.013

Intracellular metabolite concentrations are expressed as means ± S.D. (pmol/1x10^6 cells, mg protein). (+) and (-) indicate cells cultured in presence or absence of glucose, respectively.
[a] p-values were calculated using Student's t-test with significance at $P < 0.05$. Abbreviations: NAAD+, nicotinic acid adenine dinucleotide; NAADP+, nicotinic acid adenine dinucleotide phosphate; NAD+, nicotinamide adenine dinucleotide (oxidized form); NADH, nicotinamide adenine dinucleotide (reduced form); NADP+, nicotinamide adenine dinucleotide phosphate (oxidized form); NADPH, nicotinamide adenine dinucleotide phosphate (reduced form); Nam, nicotinamide; NMN, nicotinamide mononucleotide; NA, nicotinic acid; NaMN, nicotinic acid mononucleotide.

Supplementary Table S3: Significantly different gene expression levels and fold changes in parental and selected cells detected by microarray analysis

See Supplementary File: 1

Supplementary Table S4: Performance parameters for LC-MS/MS used for the quantification of compounds

ID	Retention time (min)	Ion species	Parent mass (m/z)	Product mass (m/z)	LOD[a] (ng/mL)	LLOQ[b] (ng/mL)	R^2	Fragmentor	CE (eV)	CAV (V)	Polarity
**NAAD+**	1.151	[M]+	665.0	428.0	0.5	5	0.999	380	26	5	Positive
**NAADP+**	1.142	[M]+	745.1	604.1	5	10	0.992	380	18	5	Positive
**NAD+**	1.334	[M]+	664	136	0.5	5	0.993	380	44	5	Positive
**NADH**	1.096	[M+H]+	666	649	5	10	0.998	380	12	5	Positive
**NADP+**	1.170	[M]+	744	604	5	5	0.998	380	20	5	Positive
**NADPH**	1.093	[M+H]+	746	729	5	5	0.998	380	16	5	Positive
**Nam**	1.526	[M+H]+	123	80	0.1	1	0.999	380	24	5	Positive
**NMN**	1.452	[M+H]+	335	123	0.5	1	0.992	380	10	5	Positive
**NA**	1.299	[M+H]+	124	80	0.1	0.1	0.999	380	22	5	Positive
**NaMN**	1.247	[M+H]+	336	124	0.5	1	0.991	380	20	5	Positive
**NA, ethyl ester-D4 (Internal standard)**	1.328	[M+H]+	156	83.9	-	-	-	380	32	5	Positive

Compound optimization parameters: CE, collision energy; CAV, cell accelerator voltage.

[a] Detection limits reported as the injected concentration giving a peak height corresponding to three times the baseline noise level.

[b] Lower limit of quantifications reported as the injected concentration giving a peak height corresponding to ten times the baseline noise level. Abbreviations: NAAD+, nicotinic acid adenine dinucleotide; NAADP+, nicotinic acid adenine dinucleotide phosphate; NAD+, nicotinamide adenine dinucleotide (oxidized form); NADH, nicotinamide adenine dinucleotide (reduced form); NADP+, nicotinamide adenine dinucleotide phosphate (oxidized form); NADPH, nicotinamide adenine dinucleotide phosphate (reduced form); Nam, nicotinamide; NMN, nicotinamide mononucleotide; NA, nicotinic acid; NaMN, nicotinic acid mononucleotide.
