# Supplementary material for: Integrated omics-analysis reveals Wnt-mediated NAD+ metabolic reprogramming in cancer stem-like cells
Source: Oncotarget. 2016 Jul 6;7(30):48562–76. doi: 10.18632/oncotarget.10432 (PMC5217038; doi:10.18632/oncotarget.10432)
Supplement: Supplementary file 2 [file oncotarget-07-48562-s002.docx]

**Supplementary Table 1.** Intracellular metabolites, their chemical shifts, and fold changes from ^1^H NMR spectra in parental and selected cells.

| **No.** | **ID** | **Chemical Shifts (multiplicity)*^a^*** | **(+) P-231** | **(+) S-231** | **(-) P-231** | **(-) S-231** | **Fold change** | | **p-value*^b^*** | |
| --- | --- | --- | --- | --- | --- | --- | --- | --- | --- | --- |
|  |  |  |  |  |  |  | **(+)S/(+)P** | **(-)S/(-)P** | **(+)S/(+)P** | **(-)S/(-)P** |
| 1 | **Acetate** | 1.88 (s) | 1.9 ± 1.1 | 1.6 ± 0.6 | 3.3 ± 1.2 | 2.6 ± 1.4 | 0.855 | 0.780 | 0.634 | 0.409 |
| 2 | **ADP** | 4.34 (m), 4.47 (m), 4.56 (m), 6.11 (d), 8.23 (s), 8.49 (s) | 1.3 ± 0.3 | 2.2 ± 0.6 | 4.0 ± 1.5 | 4.9 ± 1.3 | 1.678 | 1.206 | 0.021 | 0.374 |
| 3 | **Alanine** | 1.43 (d), 3.73 (q) | 2.8 ± 0.7 | 3.2 ± 1.0 | 4.1 ± 0.8 | 3.6 ± 0.9 | 1.149 | 0.866 | 0.464 | 0.338 |
| 4 | **AMP** | 4.562 (dd), 6.106 (d), 8.23 (s), 8.55 (s) | 1.1 ± 0.4 | 0.7 ± 0.3 | 4.8 ± 1.6 | 1.3 ± 0.4 | 0.632 | 0.258 | 0.120 | 0.001 |
| 5 | **Arginine** | 1.65 (m), 1.87 (m), 3.19 (t), 3.72 (t) | 4.5 ± 0.5 | 4.1 ± 0.8 | 8.6 ± 1.6 | 7.2 ± 0.9 | 0.91 | 0.835 | 0.374 | 0.130 |
| 6 | **Aspartate** | 2.65 (dd), 2.76 (dd), 3.84 (dd) | 1.8 ± 0.6 | 2.3 ± 0.9 | 11.0 ± 3.3 | 12.8 ± 1.7 | 1.294 | 1.167 | 0.324 | 0.299 |
| 7 | **Choline** | 3.16 (s), 3.48 (m), 4.02 (m) | 1.1 ± 0.2 | 1.0 ± 0.4 | 3.0 ± 0.6 | 3.1 ± 0.3 | 0.887 | 1.038 | 0.551 | 0.729 |
| 8 | **Citrate** | 2.50 (d), 2.62 (d) | 1.9 ± 0.3 | 1.8 ± 0.3 | 5.1 ± 2.3 | 4.9 ± 0.6 | 0.927 | 0.959 | 0.474 | 0.853 |
| 9 | **Glutamate** | 2.02 (m), 2.30 (m), 3.70 (m) | 31.4 ± 4.9 | 22.0 ± 6.6 | 58.3 ± 7.1 | 51.4 ± 4.3 | 0.701 | 0.882 | 0.033 | 0.100 |
| 10 | **Glutamine** | 2.11 (m), 2.40 (m), 3.72 (m) | 2.4 ± 0.6 | 3.3 ± 0.9 | 11.2 ± 2.2 | 13.8 ± 1.6 | 1.357 | 1.237 | 0.113 | 0.066 |
| 11 | **Glutathione** | 2.11 (m), 2.50 (m), 2.91 (dd), 3.74 (m), 4.52 (q) | 3.8 ± 1.0 | 2.8 ± 1.0 | 10.8 ± 2.5 | 10.8 ± 1.8 | 0.741 | 1.001 | 0.160 | 0.994 |
| 12 | **Glycine** | 3.51 (s) | 4.4 ± 1.5 | 4.0 ± 1.3 | 7.7 ± 2.3 | 6.5 ± 1.1 | 0.901 | 0.851 | 0.626 | 0.337 |
| 13 | **Histidine** | 7.12 (d), 8.03 (d) | 1.4 ± 0.4 | 1.5 ± 0.3 | 2.7 ± 0.4 | 2.7 ± 0.2 | 1.052 | 1.008 | 0.732 | 0.906 |
| 14 | **Isoleucine** | 0.89 (t), 0.96 (d), 1.22 (m), 1.44 (m), 1.91 (m), 3.62 (d) | 1.6 ± 0.4 | 1.3 ± 0.3 | 3.3 ± 0.7 | 2.9 ± 0.4 | 0.814 | 0.869 | 0.200 | 0.251 |
| 15 | **Lactate** | 1.28 (d), 4.06 (q) | 28.2 ± 7.2 | 23.9 ± 5.9 | 115.2 ± 30.2 | 73.5 ± 11.6 | 0.847 | 0.638 | 0.331 | 0.020 |
| 16 | **Leucine** | 0.91 (d), 1.65 (m), 3.66 (t) | 2.4 ± 0.6 | 2.0 ± 0.3 | 4.6 ± 0.6 | 4.1 ± 0.4 | 0.826 | 0.890 | 0.183 | 0.161 |
| 17 | **myo-Inositol** | 3.23 (t), 3.49 (dd), 3.58 (t), 4.02 (dd) | 7.8 ± 0.9 | 7.4 ± 1.3 | 10.2 ± 2.2 | 11.3 ± 2.0 | 0.957 | 1.106 | 0.639 | 0.449 |
| 18 | **NAD+** | 4.49 (m), 4.55 (m), 5.99 (d), 6.05 (d), 8.12 (s), 8.15 (m), 8.38 (s), 8.78 (d), 9.09 (d), 9.29 (s) | 5.1 ± 1.0 | 5.7 ± 1.2 | 9.8 ± 1.7 | 9.2 ± 0.9 | 1.124 | 0.939 | 0.382 | 0.496 |
| 19 | **NAAD+** | 4.417 (m), 4.519 (m), 4.774 (t), 6.00 (m), 8.01 (dd), 8.10 (s), 8.38 (s), 8.70 (d), 8.95 (d), 9.09 (s) | 3.9 ± 1.0 | 8.0 ± 1.0 | - | - | 2.084 | - | < 0.001 | - |
| 20 | **O-PC** | 3.18 (s), 3.55 (m), 4.12 (m) | 2.3 ± 0.6 | 3.5 ± 1.1 | 29.6 ± 4.5 | 38.3 ± 3.1 | 1.491 | 1.293 | 0.070 | 0.007 |
| 21 | **Phenylalanine** | 7.28 (m), 7.32 (m), 7.38 (m), | 2.0 ± 0.4 | 1.7 ± 0.3 | 3.8 ± 0.4 | 3.3 ± 0.5 | 0.824 | 0.865 | 0.135 | 0.117 |
| 22 | **Proline** | 1.96 (m), 2.30 (m), 3.30 (m), 3.36 (m), 4.09 (dd) | 18.3 ± 1.8 | 13.2 ± 1.9 | 19.9 ± 3.7 | 20.1 ± 1.5 | 0.719 | 1.008 | 0.002 | 0.933 |
| 23 | **sn-GPC** | 3.19 (s), 3.69 (dd), 3.90 (m), 4.33 (m) | 4.1 ± 1.1 | 4.0 ± 1.1 | 4.1 ± 1.1 | 3.4 ± 0.4 | 0.997 | 0.841 | 0.988 | 0.244 |
| 24 | **Succinate** | 2.36 (s) | 1.2 ± 0.3 | 0.8 ± 0.2 | 4.2 ± 1.2 | 2.7 ± 0.5 | 0.689 | 0.651 | 0.057 | 0.037 |
| 25 | **Taurine** | 3.22 (t), 3.37 (t) | 16.2 ± 4.3 | 14.0 ± 3.8 | 35.1 ± 4.3 | 34.4 ± 4.4 | 0.866 | 0.980 | 0.424 | 0.805 |
| 26 | **Tyrosine** | 6.86 (d), 7.15 (d) | 1.5 ± 0.4 | 1.4 ± 0.3 | 2.8 ± 0.5 | 2.4 ± 0.4 | 0.912 | 0.872 | 0.583 | 0.278 |
| 27 | **Uridine** | 5.86 (d), 7.83 (d) | 1.4 ± 0.5 | 1.3 ± 1.1 | 1.7 ± 1.1 | 1.1 ± .0.2 | 0.945 | 0.628 | 0.890 | 0.223 |
| 28 | **Valine** | 0.94 (d), 0.99 (d), 2.22 (m) | 1.0 ± 0.3 | 0.8 ± 0.2 | 2.2 ± .0.4 | 1.8 ± 0.3 | 0.767 | 0.826 | 0.169 | 0.128 |

Intracellular metabolite concentrations are expressed as means ± S.D. (µM/mg protein). (+) and (-) indicate cells cultured in presence or absence of glucose, respectively. *^a^*Letters in parentheses indicate the peak multiplicities: s, singlet; d, doublet; t, triplet; dd, doublet of doublet; q, quartet; and m, multiplet. *^b^*p-values were calculated using Student’s t-test with significance at P < 0.05. Abbreviations: ADP, adenosine diphosphate; AMP, adenosine monophosphate; sn-GPC, glycero-3-phosphocholine; O-PC, O-phosphocholine; NAD+, nicotinamide adenine dinucleotide (oxidized form); NAAD+, nicotinic acid adenine dinucleotide.

**Supplementary Table 3.** Significantly different gene expression levels and fold changes in parental and selected cells detected by microarray analysis.

| **Pathway** | **ID** | **(+) P-231** | **(+) S-231** | **(-) P-231** | **(-) S-231** | **Fold change** | | ***^a^*p-value** | |
| --- | --- | --- | --- | --- | --- | --- | --- | --- | --- |
|  |  |  |  |  |  | **(+)S/(+)P** | **(-)S/(-)P** | **(+)S/(+)P** | **(-)S/(-)P** |
| Ca^2+^ signaling | ADCY3 | 537.36 ± 60.64 | 422.63 ± 39.94 | 432.27 ± 42.54 | 448.16 ± 30.79 | 0.786 | 1.037 | 0.020 | 0.567 |
|  | ADCY3 | 537.36 ± 60.64 | 422.63 ± 39.94 | 432.27 ± 42.54 | 448.16 ± 30.79 | 0.786 | 1.037 | 0.020 | 0.567 |
|  | ADCY9 | 140.37 ± 6.33 | 126.3 ± 5.45 | 133.1 ± 12.61 | 118.1 ± 10.47 | 0.900 | 0.887 | 0.015 | 0.117 |
|  | ATP2A2 | 619.82 ± 34 | 744.41 ± 25.6 | 369.59 ± 32.87 | 800.6 ± 135.87 | 1.201 | 2.166 | 0.001 | 0.001 |
|  | ATP2A2 | 570.67 ± 39.74 | 657.53 ± 26.28 | 409.47 ± 30.74 | 709.19 ± 107.53 | 1.152 | 1.732 | 0.011 | 0.002 |
|  | ATP2B4 | 251.21±11.73 | 173.51±13.97 | 195.98±16.82 | 166.4±8.81 | 0.691 | 0.849 | < 0.001 | 0.021 |
|  | ATP2B4 | 221.1 ± 14.94 | 157.78 ± 16.69 | 178.35 ± 12.96 | 149.47 ± 5.93 | 0.714 | 0.838 | 0.001 | 0.007 |
|  | CALM3 | 5376.56 ± 698.57 | 3702.75 ± 250.5 | 5004.79 ± 232.76 | 4308.65 ± 385.15 | 0.689 | 0.861 | 0.004 | 0.021 |
|  | CALM3 | 5376.56 ± 698.57 | 3702.75 ± 250.5 | 5004.79 ± 232.76 | 4308.65 ± 385.15 | 0.689 | 0.861 | 0.004 | 0.021 |
|  | CALM3 | 5376.56 ± 698.57 | 3702.75 ± 250.5 | 5004.79 ± 232.76 | 4308.65 ± 385.15 | 0.689 | 0.861 | 0.004 | 0.021 |
|  | CHP | 592.01 ± 33.06 | 466 ± 38.7 | 580.57 ± 41.28 | 521.22 ± 67.35 | 0.787 | 0.898 | 0.003 | 0.184 |
|  | GNA11 | 374.58 ± 46.06 | 274.44 ± 17.06 | 331.58 ± 25.19 | 297.01 ± 23.49 | 0.733 | 0.896 | 0.007 | 0.091 |
|  | GNA15 | 1027.92 ± 178.06 | 502.68 ± 21.15 | 852.58 ± 75.47 | 545.11 ± 68.67 | 0.489 | 0.639 | 0.001 | 0.001 |
|  | GNAS | 5682.35 ± 482.18 | 4566.82 ± 443.15 | 5228.46 ± 343.19 | 5228.97 ± 378.42 | 0.804 | 1.000 | 0.014 | 0.998 |
|  | ITPKA | 233.92 ± 31.75 | 183.28 ± 5.65 | 239.55 ± 5.32 | 178.96 ± 19.61 | 0.784 | 0.747 | 0.020 | 0.001 |
|  | ITPR1 | 140.86 ± 4.44 | 124.11 ± 6.53 | 138.92 ± 6.86 | 130.96 ± 12.61 | 0.881 | 0.943 | 0.005 | 0.310 |
|  | PLCD3 | 171.79 ± 19.46 | 145.28 ± 1.78 | 145.18 ± 15.7 | 146.86 ± 12.96 | 0.846 | 1.012 | 0.035 | 0.875 |
|  | PLCG2 | 197.9 ± 18.72 | 138.83 ± 8.97 | 178.98 ± 13.04 | 142.16 ± 5.35 | 0.702 | 0.794 | 0.001 | 0.002 |
|  | PPID | 173.04 ± 22.67 | 235.08 ± 30.56 | 183.17 ± 12.36 | 240.33 ± 14.93 | 1.358 | 1.312 | 0.017 | 0.001 |
|  | PPP3R1 | 520.6 ± 79.32 | 372.62 ± 35.66 | 419.73 ± 62.29 | 397.15 ± 49.45 | 0.716 | 0.946 | 0.014 | 0.591 |
|  | PRKCA | 499.38 ± 20.01 | 437.77 ± 43.23 | 444.83 ± 66.6 | 490.66 ± 58.07 | 0.877 | 1.103 | 0.041 | 0.340 |
|  | PRKX | 112.48 ± 2.99 | 121.73 ± 3.29 | 117.01 ± 3.89 | 126.94 ± 4.87 | 1.082 | 1.085 | 0.006 | 0.019 |
|  | SLC25A5 | 6080.61 ± 755.82 | 7640.49 ± 713.13 | 6210.81 ± 590.03 | 8012.19 ± 938.54 | 1.257 | 1.29 | 0.024 | 0.017 |
| Nicotinate and nicotinamide metabolism | ENPP1 | 203.06 ± 38.76 | 131.43 ± 12.72 | 170.76 ± 19.26 | 131.97 ± 8.62 | 0.647 | 0.773 | 0.013 | 0.010 |
|  | NMNAT2 | 208.88 ± 21.26 | 140.35 ± 6.16 | 196.92 ± 11.29 | 147.39 ± 9.56 | 0.672 | 0.748 | 0.001 | 0.001 |
|  | NNT | 271.79 ± 27.14 | 207.05 ± 31.54 | 245.32 ± 20.04 | 218.06 ± 27.92 | 0.762 | 0.889 | 0.021 | 0.164 |
|  | NT5C3 | 354.06 ± 59.39 | 537.12 ± 71.1 | 447.5 ± 48.27 | 548.86 ± 100.81 | 1.517 | 1.227 | 0.008 | 0.12 |
|  | NT5C3 | 177.89 ± 21.02 | 231.22 ± 12.58 | 205.66 ± 24.65 | 245.36 ± 36.52 | 1.3 | 1.193 | 0.005 | 0.122 |
|  | NT5E | 988.07 ± 103.32 | 559.62 ± 88.75 | 1006.38 ± 122.13 | 658.48 ± 157.32 | 0.566 | 0.654 | 0.001 | 0.013 |
| Wnt signaling | CACYBP | 119.98 ± 10.43 | 150.11 ± 12.57 | 130.35 ± 7.83 | 149.4 ± 6.55 | 1.251 | 1.146 | 0.010 | 0.010 |
|  | CACYBP | 1426.27 ± 265.69 | 1937.66 ± 163.96 | 1912.77 ± 94.95 | 1658.98 ± 212.45 | 1.359 | 0.867 | 0.017 | 0.072 |
|  | CCND3 | 1483.48 ± 302.41 | 1963.2 ± 54.8 | 1699.97 ± 110.91 | 2062.82 ± 242.98 | 1.323 | 1.213 | 0.021 | 0.035 |
|  | CHP | 592.01 ± 33.06 | 466 ± 38.7 | 580.57 ± 41.28 | 521.22 ± 67.35 | 0.787 | 0.898 | 0.003 | 0.184 |
|  | CSNK1A1 | 226.14 ± 18.48 | 265.71 ± 14.76 | 255.88 ± 17.7 | 262.1 ± 46.5 | 1.175 | 1.024 | 0.015 | 0.811 |
|  | CSNK1A1 | 111.79 ± 4.68 | 123.94 ± 3.1 | 117.21 ± 6.45 | 131.44 ± 10.7 | 1.109 | 1.121 | 0.005 | 0.063 |
|  | CSNK1E | 1626.19 ± 182.6 | 1299.85 ± 61.71 | 1603.33 ± 95.97 | 1481.29 ± 209.04 | 0.799 | 0.924 | 0.015 | 0.329 |
|  | CSNK1E | 275.33 ± 24.94 | 223.25 ± 22.28 | 236.9 ± 17.47 | 265.65 ± 34.59 | 0.811 | 1.121 | 0.021 | 0.188 |
|  | CSNK2B | 1437.66 ± 225.79 | 1888.23 ± 207.77 | 1368.29 ± 119.72 | 1998.2 ± 195.32 | 1.313 | 1.46 | 0.026 | 0.002 |
|  | CTNNBIP1 | 191.31±10.24 | 138.53±11.19 | 158.39±6.16 | 151.15±5.74 | 0.724 | 0.954 | < 0.001 | 0.136 |
|  | DAAM1 | 123.76±12.98 | 206.7±17.09 | 138.14±8.26 | 230.13±17.12 | 1.67 | 1.666 | < 0.001 | < 0.001 |
|  | DVL2 | 181.08 ± 14.04 | 202.57 ± 5.13 | 168.33 ± 7.89 | 222.78 ± 24.99 | 1.119 | 1.323 | 0.028 | 0.006 |
|  | FBXW11 | 230.65 ± 23.65 | 187.32 ± 8.69 | 182.57 ± 13.94 | 200.72 ± 19.41 | 0.812 | 1.099 | 0.014 | 0.18 |
|  | FBXW11 | 510.44 ± 46.48 | 413.86 ± 48.82 | 630.84 ± 123.06 | 470.91 ± 54.12 | 0.811 | 0.746 | 0.029 | 0.055 |
|  | FZD4 | 147.05 ± 12.68 | 125.07 ± 7.02 | 143.42 ± 4.73 | 120.2 ± 6.03 | 0.851 | 0.838 | 0.023 | 0.001 |
|  | FZD7 | 213.7 ± 21.85 | 138.08 ± 10.5 | 198.67 ± 14.39 | 146.03 ± 12.62 | 0.646 | 0.735 | 0.001 | 0.002 |
|  | GSK3B | 271.38 ± 34.61 | 372.52 ± 45.19 | 290.02 ± 15.59 | 353.53 ± 38.3 | 1.373 | 1.219 | 0.012 | 0.022 |
|  | LRP5 | 438.29 ± 63.09 | 228.48 ± 16.12 | 264.24 ± 27.26 | 271.29 ± 26.53 | 0.521 | 1.027 | 0.001 | 0.723 |
|  | MYC | 939.99 ± 122.72 | 1182.89 ± 77.05 | 775.5 ± 73.34 | 1286.03 ± 186.83 | 1.258 | 1.658 | 0.015 | 0.002 |
|  | MYC | 336.4 ± 73.19 | 439.54 ± 36.09 | 320.6 ± 45.19 | 482.77 ± 100.62 | 1.307 | 1.506 | 0.045 | 0.026 |
|  | PPARD | 125.91 ± 7.29 | 139.08 ± 1.65 | 116.03 ± 6.37 | 151.82 ± 8.24 | 1.105 | 1.309 | 0.012 | < 0.001 |
|  | PPP2CB | 113.4 ± 7.15 | 125.2 ± 5.91 | 124.34 ± 6.53 | 118.96 ± 5.59 | 1.104 | 0.957 | 0.044 | 0.257 |
|  | PPP2R1A | 1315.36 ± 234.2 | 804.1 ± 64.91 | 1110.41 ± 107.82 | 943.96 ± 123.96 | 0.611 | 0.850 | 0.006 | 0.089 |
|  | PPP2R5C | 289.02 ± 56.47 | 388.15 ± 18.34 | 350.55 ± 23.7 | 464.69 ± 57.01 | 1.343 | 1.326 | 0.016 | 0.010 |
|  | PPP3R1 | 520.6 ± 79.32 | 372.62 ± 35.66 | 419.73 ± 62.29 | 397.15 ± 49.45 | 0.716 | 0.946 | 0.014 | 0.591 |
|  | PRICKLE2 | 188.74 ± 19.17 | 129.76 ± 11.17 | 176.01 ± 8.99 | 137.03 ± 14.36 | 0.688 | 0.779 | 0.002 | 0.004 |
|  | PRKCA | 499.38 ± 20.01 | 437.77 ± 43.23 | 444.83 ± 66.6 | 490.66 ± 58.07 | 0.877 | 1.103 | 0.041 | 0.340 |
|  | PRKX | 112.48 ± 2.99 | 121.73 ± 3.29 | 117.01 ± 3.89 | 126.94 ± 4.87 | 1.082 | 1.085 | 0.006 | 0.019 |
|  | RAC2 | 2803.18 ± 421.72 | 2072.06 ± 157.68 | 2766.62 ± 186.69 | 2364.81 ± 331.32 | 0.739 | 0.855 | 0.018 | 0.079 |
|  | RHOA | 2186.6 ± 314.69 | 1570.4 ± 140.44 | 2259.9 ± 217.14 | 1808.24 ± 270.62 | 0.718 | 0.800 | 0.012 | 0.040 |
|  | SIAH1 | 218.94 ± 29.89 | 273.57 ± 27.52 | 239.79 ± 12.14 | 306.74 ± 30.27 | 1.250 | 1.279 | 0.036 | 0.006 |
|  | SMAD4 | 215.45 ± 11.14 | 273.69 ± 22.93 | 210.55 ± 13.9 | 290.59 ± 39.41 | 1.270 | 1.38 | 0.004 | 0.009 |
|  | TCF3 | 303.05 ± 34.24 | 220.81 ± 18.79 | 263.13 ± 12.76 | 216.83 ± 26.81 | 0.729 | 0.824 | 0.006 | 0.021 |
|  | TP53 | 193.17 ± 20.8 | 160.49 ± 6.52 | 171.71 ± 14.66 | 178.4 ± 15.8 | 0.831 | 1.039 | 0.024 | 0.557 |
|  | WNT5B | 119.66±10.04 | 159.43±6.01 | 119.05±8.48 | 176.32±16.39 | 1.332 | 1.481 | < 0.001 | 0.001 |

(+) and (-) indicate cells cultured in presence or absence of glucose, respectively. *^a^*p-values were calculated using Student's t-test with significance at P < 0.05.
